# Seasonal dynamics in sheep fecal microbiome and soil bacterial communities under grazing management

**DOI:** 10.1371/journal.pone.0352436

**Published:** 2026-06-29

**Authors:** Maria Chiara Fabbri, Iliyass Biada, Maria Teresa Ceccherini, Christian Maltecca, Beatrice Fiore, Francesco Sirtori, Lina Pulido-Rodríguez, Giovanni Mastrolonardo, Riccardo Bozzi, Francesco Tiezzi

**Affiliations:** 1 Department of Agriculture, Food, Environment, and Forestry (DAGRI), University of Florence, Florence, Italy; 2 Institute for Animal Science and Technology, Universitat Politècnica de València, Valencia, Spain; 3 Department of Animal Science, North Carolina State University, Raleigh, North Carolina, United States of America; Institute for Sustainable Plant Protection, C.N.R., ITALY

## Abstract

The gut microbiome plays a key role in animal health, productivity, and environmental sustainability. As it represents a valuable proxy for animal welfare, its investigation has become increasingly important in livestock studies. With the growing focus on promoting sustainable livestock, supporting rural areas at risk of abandonment is receiving particular attention. Indeed, sheep grazing offers a promising strategy for improving sustainability, biodiversity, and land management. This study focuses on the interconnected dynamics between the sheep gut and soil microbiomes, assessing how seasonal changes and grazing activity shape microbial diversity and community structure across the animal–soil interface. Fecal and soil samples were collected throughout 2024 in a commercial farm in Tuscany, Italy: 215 fecal and 46 soil samples (23 pasture and 23 meadow – i.e., not grazed) were stored. Alpha and Beta diversity were assessed using the Kruskal-Wallis test and PERMANOVA, respectively, and the differential abundance analysis was also performed. The relative abundance analysis at the family and genus level revealed an increase in the number of taxa from winter to autumn in both fecal and soil samples. When the Chao1 index was considered, alpha diversity was higher in fecal samples, followed by soils. Principal Coordinate Analysis revealed distinct clustering between animal and soil microbiota, with slightly reduced differentiation in Summer. In fecal samples, the five most abundant bacterial families were *Ruminococcaceae*, *Spirochaetaceae*, *Porphyromonadaceae*, *Lachnospiraceae*, and *Rikenellaceae*, whose abundance varied seasonally. *Ruminococcaceae, Lachnospiraceae,* and *Rikenellaceae* decreased in Summer, while *Spirochaetaceae* and *Porphyromonadaceae* increased. The increased abundance of these families during Summer may reflect heat stress in animals. Differential abundance analysis also suggested potential microbial transfer from animals to soil: *Peptostreptococcaceae* and *Erysipelotrichaceae* were enriched in grazed soils across multiple seasons. Repeated cross-sectional studies like this are essential for understanding microbiome dynamics and animal–soil interactions in grazing systems.

## Introduction

The ruminant gut microbiome is fundamental to animal health, productivity, and overall fitness. Ruminants rely on a complex and dense microbial community in the rumen, composed of bacteria, archaea, protozoa, and fungi, to break down plant polysaccharides, such as cellulose and hemicellulose compounds, indigestible by the host’s own enzymes [1–3]. This fermentation process produces volatile fatty acids (VFAs), which supply up to 70% of the animal’s energy needs and are essential for metabolic functions, including fatty acid synthesis and gluconeogenesis [1]. The microbiota also contributes to the synthesis of microbial proteins, crucial for the animal’s growth and milk production, while supporting immune development and protecting against pathogens [4]. Variations in microbial gut composition can significantly influence feed efficiency, nutrient absorption, and the quality of animal products such as milk and meat [2,4]. Therefore, understanding the ruminant gut microbiota is important for optimizing animal fitness, improving production efficiency, and developing strategies to reduce environmental impacts from livestock farming [2,4].

Sampling the gut and rumen microbiome often requires expensive and invasive — or not that effective — sampling methods [5]. These include: rumen cannulation or stomach tubing, which can cause stress to the animal; collection of post mortem gut contents and mucosal scrapings, which precludes over‑time studies [3]; endoscopic or biopsy-based sampling, which is technically challenging in large animals and often requires anesthesia or surgical intervention [3,5]. Fecal sampling, on the other hand, is non-invasive, simple, and repeatable. This ease of collection allows for large-scale and longitudinal/cross-sectional studies without compromising animal welfare. Although the fecal microbiome does not perfectly mirror the rumen microbial community, it reliably reflects the microbial ecology of the lower gut, making fecal sampling a practical and less invasive method to study the distal gut environment [5], supporting its use as a proxy for animal health, nutritional status, and responses to environmental stressors.

One clear environmental stressor is the heat experienced by ruminants during late spring and summer. Describing the animals’ physiological stress and identifying specific changes in microbial community composition, particularly at lower taxonomic levels, is important for understanding microbiota responses to heat stress. Such insights may also support the development of mitigation strategies, such as dietary interventions and environmental modifications, to preserve animal health and performance [6].

Few studies have investigated the microbiota of sheep raised under grazing systems, likely because microbial communities are influenced by a complex interplay of factors, such as diet, season, lactation stage, parity, and breed. Nonetheless, this type of research is essential, as it allows us to assess how grazing influences soil microbiota and, in turn, how soil microbial communities affect grazing systems. Sheep grazing can alter the structure, diversity, and function of soil microbial communities, depending on grazing intensity, management system, and grassland condition. Sustainable grazing has been shown to positively impact soil microbial biomass and activity, thereby enhancing soil structure and overall ecosystem functioning [7,8]. On the other hand, high grazing intensity may deplete soil organic carbon and shift microbial composition by reducing or promoting specific bacterial and fungal taxa [9].

Limited evidence connects these soil changes to the sheep gut microbiota. However, a shift from non‐grazing to grazing diets increases rumen microbial diversity and network complexity [10]. Understanding these microbiome interactions may provide valuable insight into soil health and animal–soil relationships in grazing systems, thereby contributing to the biological knowledge needed to support sustainable grazing practices. In a broader context, at a time when innovative strategies are urgently needed to promote sustainability, improve animal welfare, and prevent the abandonment of marginal rural areas, extensive sheep grazing may represent a promising approach that could contribute to these goals [11].

This study aims to provide an initial investigation of seasonal variations in the taxonomic composition of the sheep gut microbiota and to evaluate the effects of grazing on soil microbial dynamics, with a primary focus on the bacterial community. To this end, fecal and soil samples were collected across all seasons of 2024 at a commercial farm in Tuscany, Italy.

## Materials and methods

### Experimental design, localization, and soil characterization

**Fig 1:**
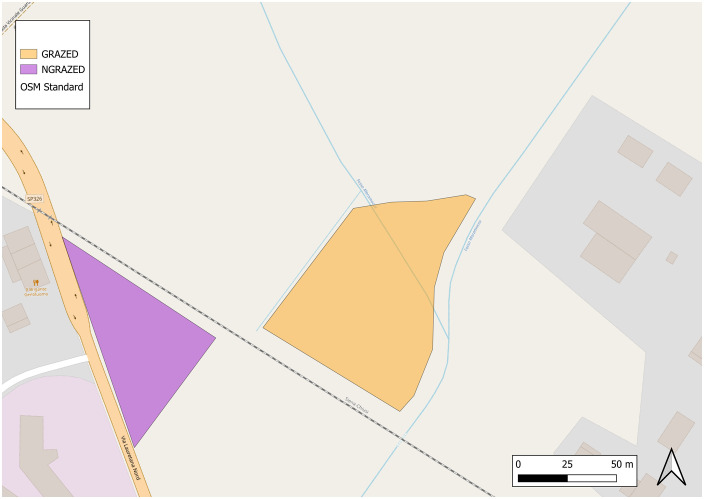
Representation of the pilot farm and sampling sites of the study, where grazed soil (GRAZED) is in orange, and the not-grazed soil (NGRAZED) is in purple.

Animals and soil samples were collected in a farm in Torrita di Siena, Tuscany, Italy (43.182926, 11.773025), showed in Fig 1. Maps of the sampling areas (i.e., grazed and ungrazed) were created using QGIS version 3.44 [12], based on data from OpenStreetMap (© OpenStreetMap contributors), which are available under the Open Database License (ODbL). Field site access and sampling permissions were granted by the owners and managers of the farm where the study was conducted. The climate of Torrita di Siena is temperate subcontinental, characterized by short, warm, mostly clear summers and long, cold, partly cloudy winters. Annual temperatures generally range from 0 °C to 31 °C, rarely dropping below –5 °C or exceeding 35 °C. The warm season lasts about three months, with mean daily highs above 26 °C, peaking at around 30 °C in midsummer, while the cool season extends from mid-November to early March, with mean highs below 13 °C and lows near freezing. Precipitation occurs throughout the year, but peaks in autumn (average ~87 mm in the wettest month) and reaches its minimum in summer (about 23 mm in the driest month) [13].

The study area is characterized by gently undulating surfaces developed on colluvial deposits composed of undifferentiated clayey, silty, and sandy sediments accumulated at the foot of hilly slopes as a result of gravity and surface runoff from rainfall. These deposits gradually thin out and disappear in the adjacent plain areas. The soils, classified as Vertic Luvisols, are generally deep, slightly gravelly, with a silt loam texture at the surface and a silty clay loam texture in depth, with an Ap-Bt-2Btk horizons sequence [14]. During each sampling period, approximately 120–150 ewes were simultaneously in lactation. This number varied slightly because animals were grouped according to their lactation stage as part of routine herd management. Sheep were allowed to graze, roaming free within fenced areas every day, for a variable amount of time depending on the season, but approximately 3 hours per day.

**Fig 2:**
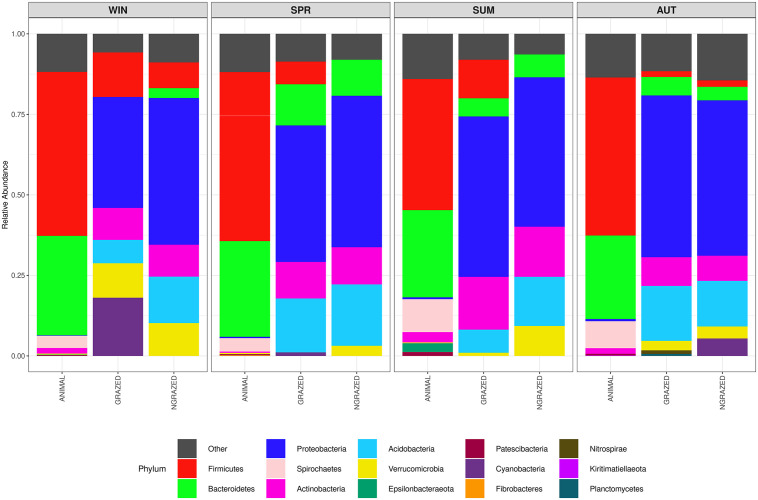
The phylum-level microbial composition of ANIMAL, GRAZED, and NGRAZED groups divided by season.

Sampling was conducted at two sites: 1) grazed soil (GRAZED — colored orange in Fig 1), and 2) a nearby plot never accessed by animals, the not-grazed soil (NGRAZED — colored purple in Fig 1). GRAZED was a grass pasture, while NGRAZED was an uncultivated meadow, which is clipped periodically for the production of hay.

For the physical characterization of the soil, five bulk soil samples were collected using a volumetric cylinder measuring 10 cm in height and 4.4 cm in diameter. In each area, a 12-meter by 12-meter square was identified, from which five samples were collected (one from each corner and one from the center).

The sampling cylinder was inserted into the soil, and the obtained samples were then weighed, placed in a container, and transferred to an oven at 105°C for at least 24 hours to completely remove moisture. Once a constant weight was reached, the sample was reweighed using an analytical balance.

This method allows for an accurate determination of soil bulk density (BD), a fundamental parameter for describing the physical structure of the soil. BD is defined as the ratio between the dry mass of the soil and the total volume it occupies, including both the solid phase and pore spaces. BD values were used to evaluate soil physical status and to assess the impact of grazing on soil structure.

Measurements of pH and electrical conductivity (EC) were carried out in a 1:2.5 soil-to-distilled water suspension using an XS PC8 pH/EC meter. Soil particle-size distribution was determined using the densiometric method, dispersing the particles with a sodium hexametaphosphate solution (5% w/v). The suspension was then mechanically stirred and allowed to settle, and particle-size fractions (sand, silt, and clay) were determined according to Stokes’ law by measuring suspension density at specified time intervals with a hydrometer. This variable was determined on a pooled sample obtained by combining all individual soil samples, and the analysis was performed in laboratory duplicate. Results were expressed as the percentage of each fraction relative to the total sample weight. Organic matter content was determined using the Loss on Ignition (LOI) method, involving the combustion of the soil samples at 470°C for a four-hour duration to ensure complete oxidation of the organic matter. The organic matter content was calculated as the percentage of mass loss relative to the initial weight of the sample dried at 105°C.

### Animal and soil bacteriome samples collection

The sampling of animals was approved by the OPBA (i.e., Animal Welfare Organization) of the University of Florence (Protocol number 0226378), by Directive 2010/63/EU of the European Parliament and the Council of 22 September 2010, updating Directive 86/609/EEC on the protection of animals used for scientific purposes. Lactating ewes were chosen as study population because they represent a metabolically homogeneous and management-relevant group within the flock.

Soil and sheep fecal samples were collected once each season throughout 2024, all samples represent biological replicates, with no technical replicates included. Soil samples were collected with a trowel from multiple spatially distributed points within the GRAZED and NGRAZED perimeters, in equal numbers within each season, avoiding adjacent sampling locations and collecting bulk soil at a depth of 10 cm (the number of soil samples varied across seasons because the sampling effort was progressively expanded as additional funding became available during the study). Fecal swabs (FLOQSwabs®,Copan, Italy) were used to sample animals’ microbiota, and the collection was only carried out during milking, in order to minimize animals’ stress. In total, 215 lactating sheep were sampled, 50 in Winter, 54 in Spring, 70 in Summer, and 41 in Autumn. The number of soil samples was 46: 6 in Winter, 12 in Spring and Summer, and 16 in Autumn. All samples were stored in a dry ice box immediately after collection and transfer to the laboratory, then stored at – 80°C until further analysis.

### Sequencing, bioinformatic, and statistical analyses

DNA was extracted using the Mag-Bind Universal pathogen kit – Omega Bio-tek. Bacterial genomic DNA was amplified and purified following the Illumina protocol “16S Metagenomic Sequencing Library Preparation” by an external lab. In detail, the V3-V4 region of the 16S rRNA was amplified utilizing specific primers (16S-341forward: 5’ – TCGTCGGCAGCGTCAGATGTGTATAAGAGACAGCCTACGGGNGGCWGCAG – 3’ and 16S-805 reverse: 5’- GTCTCGTGGGCTCGGAGATGTGTATAAGAGACAGGACTACHVGGGTATCTAATCC – 3’) with the suggested cycling conditions: 25 cycles of 95°C for 30 s, 55°C for 30 s, 72°C for 30 s and a final extension at 72°C for 5 min. Quality control and quantification of the libraries were performed using the Fragment Analyzer system (Agilent Technologies). Libraries were then normalized to a specific molarity and pooled. Sequencing was carried out on an Illumina Nextseq2000 sequencer (Illumina, United States), producing paired-end reads of 300 bp.

Bioinformatic elaborations were performed in R v4.4.3 using DADA2 package [15], version 1.34.0. According to the quality profiles, forward reads were retained at their full length (300 bases), and reverse reads were truncated at 280 bases. To eliminate adapters, the first 20 bases were removed from both the forward and the reverse reads. Specific error rates were estimated for the forward reads and for the reverse reads and then used to infer the Amplicon Sequence Variants (ASVs) [16], and the read pairs were merged with default parameters. Chimeric sequences were removed using the consensus method. Data were filtered to exclude non-bacterial ASVs, ASVs with an overall frequency less than 0.01%, and ASVs with a sample prevalence < less than 10%. Pruning was applied separately to soil and fecal datasets: ASVs were retained if present in at least 21 fecal samples (animals) or 5 soil samples. No rarefaction was performed. Taxonomic assignment of sequence variants was performed using the pre-trained SILVA classifier v.132 [17]. Amplicon sequence variant (ASV) characterization and downstream statistical analyses were conducted in R with the ‘vegan’ package v.2.6–10 [18]; relative abundances were calculated for all retained ASVs.

Physico-chemical data for GRAZED and NGRAZED soils were checked for normal distribution using the Shapiro–Wilk test and compared using a two-tailed independent-samples t-test at a 95% confidence level using SPSS software, version 29 (IBM Corp., Armonk, NY, USA).

### Alpha and beta diversity indexes

Alpha diversity was calculated using Shannon and Chao1 diversity indexes. To assess differences in intra-sample diversity among groups (ANIMAL, GRAZED, and NGRAZED), the Kruskal–Wallis test was applied separately within each season (Winter, Spring, Summer, and Autumn). When significant effects were detected, Dunn’s post hoc test was used to perform pairwise comparisons among groups within each season. Beta diversity was computed using Bray–Curtis distance matrices and visualized through principal coordinates analysis (PCoA), performed with the ecodist R package v.2.1.3 [19].

Bray–Curtis dissimilarities were also evaluated non-parametrically via permutational analysis of variance (PERMANOVA) by using 10,000 permutations. Each season was tested separately, and the pairwise contrasts between ANIMAL, NGRAZED, and GRAZED were obtained using the pairwiseAdonis R package v.0.4.1 [20]. To assess the assumption of homogeneity of multivariate dispersion, PERMDISP analysis was performed using the *betadisper* function in the vegan R package, followed by permutation tests.

Differential abundance analysis was performed with the DESeq2 R package v.1.36 [21], and results were visualized through volcano plots with the EnhancedVolcano R package v.1.24 [22]. Differential taxa were identified using the Wald test implemented in DESeq2. Statistical significance was primarily determined using the Benjamini–Hochberg false discovery rate (FDR) with a threshold of 0.05, combined with a fold-change (FC) threshold higher than 1.5 or lower than −1.5 to ensure biological relevance. In addition, Bonferroni-adjusted p-value thresholds were applied as a more conservative filter to highlight the most robust differences in the results. Because a substantially larger number of significant taxa was detected in the ANIMAL dataset compared to soil samples, a more stringent threshold was applied for ANIMAL comparisons (adjusted p ≤ 1 × 10 ⁻ ²⁰) than for soil comparisons (adjusted p ≤ 1 × 10 ⁻ ²), in order to facilitate visualization and interpretation of the most strongly differentiated taxa.

## Results

### Experimental design, localization, and soil characterization

The basic physico-chemical characterization of soils is described in Table 1 (raw data reported in S1 Table).

**Table 1:** Main physico-chemical characteristics of the soil samples collected in the GRAZED and NGRAZED areas. Values are reported as the mean of five independent replicates (in parentheses standard deviations). Soil texture was determined on a single pooled sample.

	Bulk Density (g/cm³)	pH	EC (µS/cm)	Soil Organic Matter (%)	Sand (%)	Silt (%)	Clay (%)
GRAZED	1.27 (0.07)*	6.78 (0.28)	1.68 (0.28)	5.44 (0.40)	2	84	14
NGRAZED	1.09 (0.12)*	6.77 (0.28)	1.73 (0.28)	5.33 (0.26)	3	79	18

* significant differences between GRAZED and NGRAZED areas (p < 0.05).

Soil texture was uniform in both the investigated areas and classified as silt loam, being composed of approximately 80% silt, 15–20% clay, and the remaining fraction of sand. The Bulk Density and SOM values obtained in the investigated areas were below 1.3 g/cm³ and above 5%, respectively, generally indicative of good soil structure, even if GRAZED soil was significantly more compacted than NGRAZED one. The EC values of the sampled soils ranged from 1.68 to 1.73 µS/cm, while pH values ranged from 6.77 to 6.78. These values fall within the neutral range and are considered optimal for soil biological activity.

### Taxonomic composition of animal and soil microbiomes

After filtering, 3 animals (belonging to autumn sampling) have been excluded from the analysis due to the low number of reads; 212 animals were included in all further analyses. The final feature tables used for statistical analyses had 1,500 ASVs, with a total of 17,994,636, 3,218,436, and 2,570,827 reads for ANIMAL, GRAZED, and NGRAZED, respectively. The mean (and sd) read count per sample used was 83,695 (±28,338) in ANIMAL, 139,932 (±48,810) in GRAZED, and 111,775 (±69,179) in NGRAZED samples.

**Fig 3:**
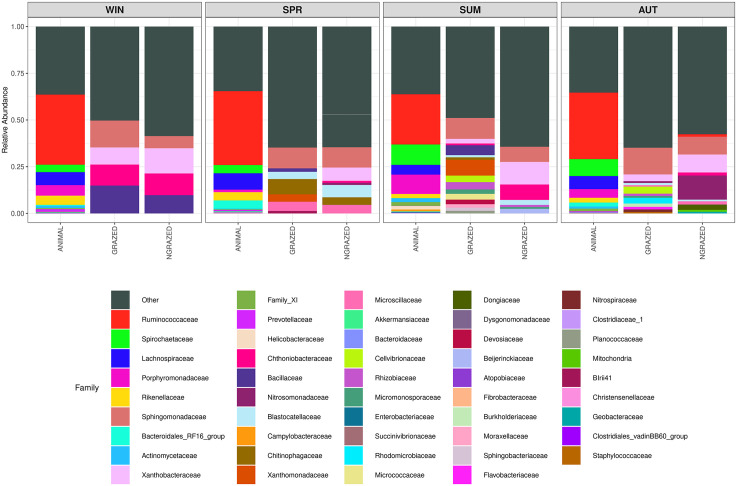
The family-level bacterial composition of each group in each season.

**Fig 4:**
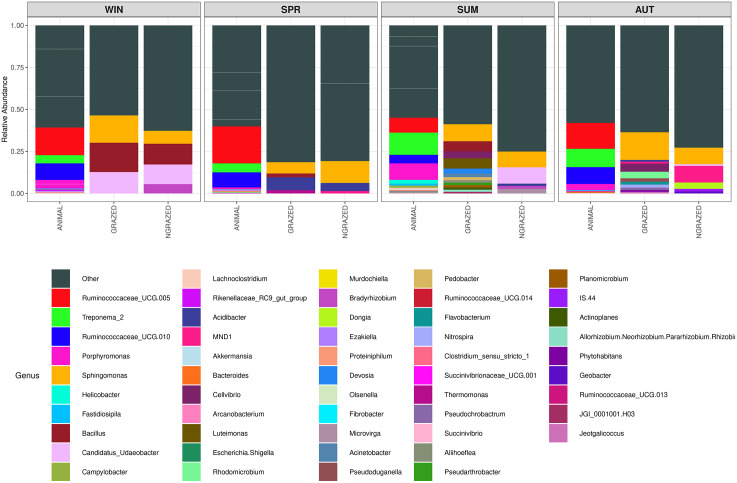
The genus-level microbial composition of each group in each season.

The relative abundance of the bacteriome for each group is presented at the phylum level in Fig 2, at the family level in Fig 3, and at the genus level in Fig 4 (more details are provided in S2 Table). In animal samples, a total of 22 phyla were identified, of which 14 showed a relative abundance greater than 5%. The most representative phyla in the animal group were *Firmicutes, Bacteroidetes*, and *Spirochaetes*. Seasonal variation slightly influenced phylum-level composition, with *Epsilonbacteraeota* detected exclusively in Summer ANIMAL samples and an increase in *Spirochaetes* observed from Spring through Summer.

The dominant phyla in soil were *Proteobacteria*, *Actinobacteria*, *Acidobacteria*, and *Verrucomicrobia*. *Firmicutes* were prominent in Winter across both soil groups, while in Spring and Summer, they were detected only in GRAZED samples; in Autumn, they were also present in NGRAZED soils. *Actinobacteria* were less abundant in animal samples compared to soils, whereas *Acidobacteria* were found exclusively in soil across all seasons. *Cyanobacteria* were detected in GRAZED soils, showing a decreasing trend from Winter to Autumn. This decline coincided with the emergence of *Bacteroidetes* in Spring and *Verrucomicrobia* in Summer. In NGRAZED soils, *Cyanobacteria* were detected only in Autumn (Fig 2).

Globally, 119 families were classified, 48 with an abundance higher than 5% (23 in ANIMAL, 25 in GRAZED, 17 in NGRAZED). The five most abundant families in the ANIMAL group were *Ruminococcaceae*, *Spirochaetaceae*, *Porphyromonadaceae*, *Lachnospiraceae,* and *Rikenellaceae* (Fig 3). These families changed their abundance based on the season. In summer, the relative abundance of *Ruminococcaceae* decreased, whereas *Spirochaetaceae* and *Porphyromonadaceae* increased, together with a concurrent reduction in *Lachnospiraceae* and *Rikenellaceae.* The bacterial composition of GRAZED and NGRAZED differed between the two plots and varied across seasons. The most abundant families in Winter were: *Bacillaceae*, *Chthoniobacteraceae*, *Sphingomonadaceae,* and *Xanthobacteraceae*. From Spring to Autumn, the grazed soil was characterized by a more diversified bacterial composition, where *Sphingomonadaceae* remained the most abundant Family. Also, for NGRAZED, the variability increased from Spring to Autumn, but the most recurrent Family was the *Xanthobacteraceae*.

At the genus level, 170 genera were identified, 53 of which had a relative abundance of at least 5% (Fig 4). In ANIMAL, the presence of *Treponema_2*, belonging to the *Spirochaetaceae* family, was particularly noteworthy, as it was present only in this sample, and increased in abundance during the Summer. Additionally, *Porphyromonas* nearly decreased in Spring and Autumn but reemerged consistently in Summer. *Ruminococcaceae family* was detectable only in ANIMAL samples with a percentage higher than 5%.

In Winter, *Candidatus_Udaeobacter*, *Sphingomonas,* and *Bacillus* were the most abundant genera in both soils; from Spring to Autumn, the relative abundance changed differently in the two groups, showing a higher bacterial number of taxa in GRAZED than in NGRAZED, especially in Summer and Autumn.

### Alpha and Beta Diversity Indices

**Fig 5:**
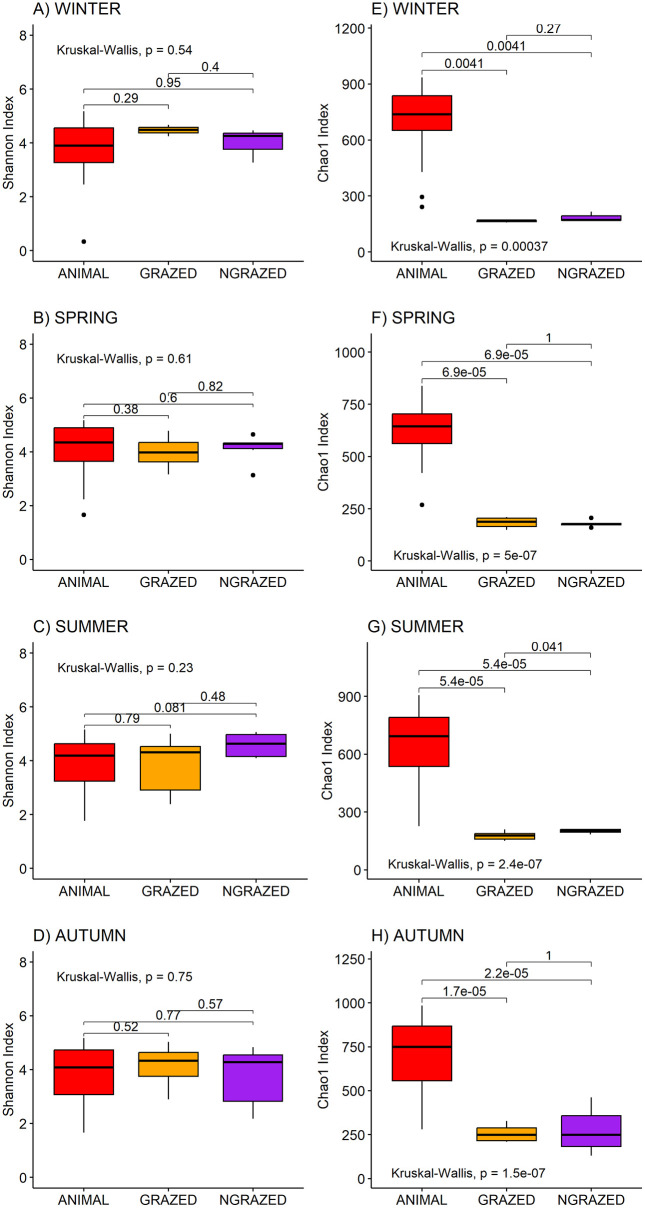
Box plots representing the alpha diversity indexes (Shannon – A, B, C, D, and Chao1 – E, F, G,H), and the related significant differences obtained through the Dunn test (p ≤ 0.05).

Two alpha-diversity measures were evaluated: the Shannon index and the Chao1 index. Overall, the Shannon index showed similar levels of diversity across all samples among seasons, while Chao1 showed variations in diversity among seasons (Fig 5).

Based on Chao1, fecal samples exhibited significantly higher alpha diversity than both GRAZED and NGRAZED across all seasons. Mean Chao1 values were 676 ± 155 for ANIMAL, 218 ± 81 for NGRAZED, and 204 ± 49 for GRAZED. Within ANIMAL, Chao1 was highest in Winter (724) and lowest in Spring (631); for NGRAZED and GRAZED, the highest seasonal values were in Autumn (274 and 256, respectively).

For Shannon, mean values were 3.97 ± 0.88 (ANIMAL), 4.10 ± 0.79 (NGRAZED), and 4.07 ± 0.75 (GRAZED). In ANIMAL, Shannon was highest in Spring (4.18) and lowest in Autumn (3.82); NGRAZED peaked in Summer (4.59) and GRAZED in Winter (4.47).

**Fig 6:**
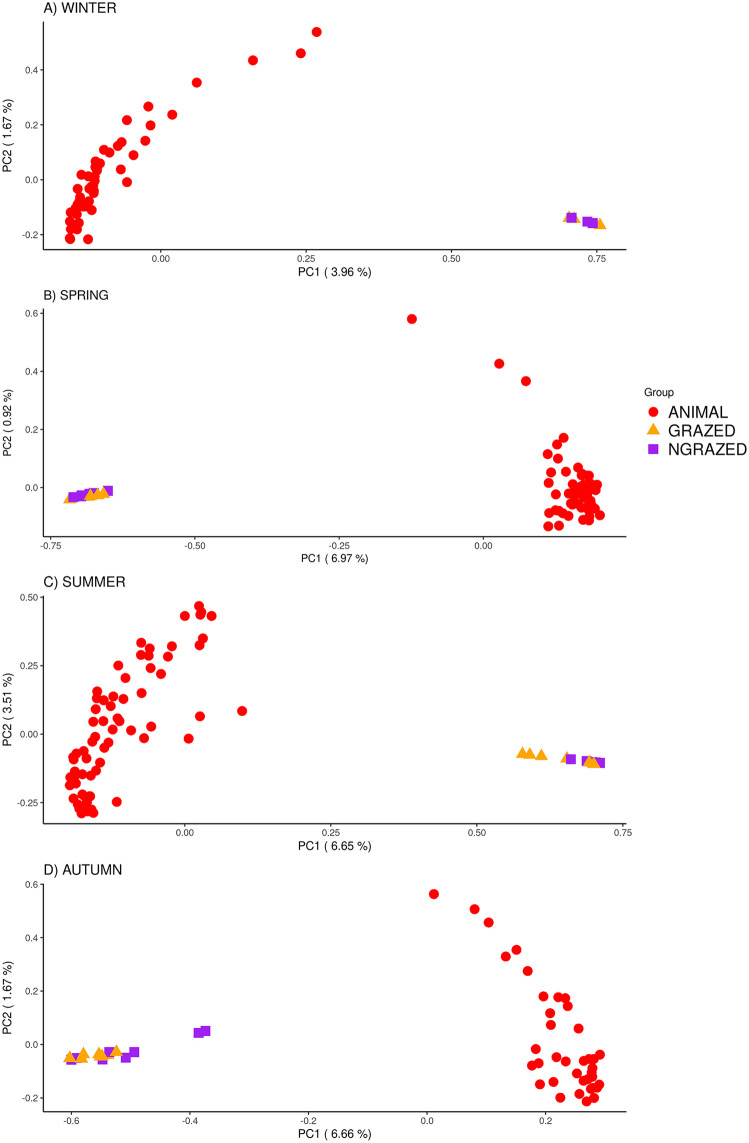
PCoA plots depicting the distribution of groups (ANIMAL, GRAZED, NGRAZED) for each season using the Bray–Curtis distances.

Beta diversity was tested using two different approaches: PERMANOVA (Table 2) and Principal Coordinate Analysis with Bray-Curtis distances (Fig 6). The microbial communities in animal and soil samples were significantly different.

**Table 2:** PERMANOVA testing the effect of sample group (ANIMAL, GRAZED, NGRAZED) on microbial community composition within each season.

		Df	Sum Sq	R^2^	F	P
WINTER	Group	2	4.01	0.34	13.74	0.0001
	Residual	53	7.82	0.66		
	Total	55	11.88	1.00		
SPRING	Group	2	7.31	0.44	24.50	0.0001
	Residual	63	9.40	0.56		
	Total	65	16.71	1.00		
SUMMER	Group	2	7.21	0.33	19.21	0.0001
	Residual	79	14.83	0.67		
	Total	81	22.04	1.00		
AUTUMN	Group	2	7.13	0.44	19.95	0.0001
	Residual	51	9.12	0.56		
	Total	53	16.25	1.00		

Df = Degrees of Freedom; Sum Sq = Sum of Squares; R² = proportion of variation explained; F = F statistic; P = permutation p-value; Residual = variation not explained by the group factor.

In particular, the PERMANOVA analysis highlighted significant differences for groups in each season, as well as for all pairwise comparisons across all levels, with the exception of the contrast revealed in GRAZED vs NGRAZED in Winter (p value > 0.05, S3 Table). The assumption of homogeneity of multivariate dispersion was tested using PERMDISP. No significant differences among groups were observed in Winter (p = 0.19) and Autumn (p = 0.45). In contrast, significant differences in dispersion were detected in Spring (p = 0.008) and Summer (p = 0.001). These results suggest that, for Spring and Summer, PERMANOVA outcomes may be partially influenced by differences in within-group variability and should therefore be interpreted with caution.

The PCoA plot clearly showed two distinct clusters: animals and soil, which are well separated. It also revealed high variability within animal samples across all seasons, which compressed the soil samples and made it difficult to distinguish between GRAZED and NGRAZED soils. In general, the combined dataset including both sheep fecal and soil samples amplified the heterogeneous microbial environments. This contributed to the dispersion of variance across multiple axes and this explains why the percentages explained for each axis is low. However, in Summer, GRAZED soil samples appeared closer to animal samples compared to other seasons, the same for few NGRAZED samples in Autumn. Zoomed-in figures for soil samples are provided in the S1 Fig.

### Differential abundance analysis

**Fig 7:**
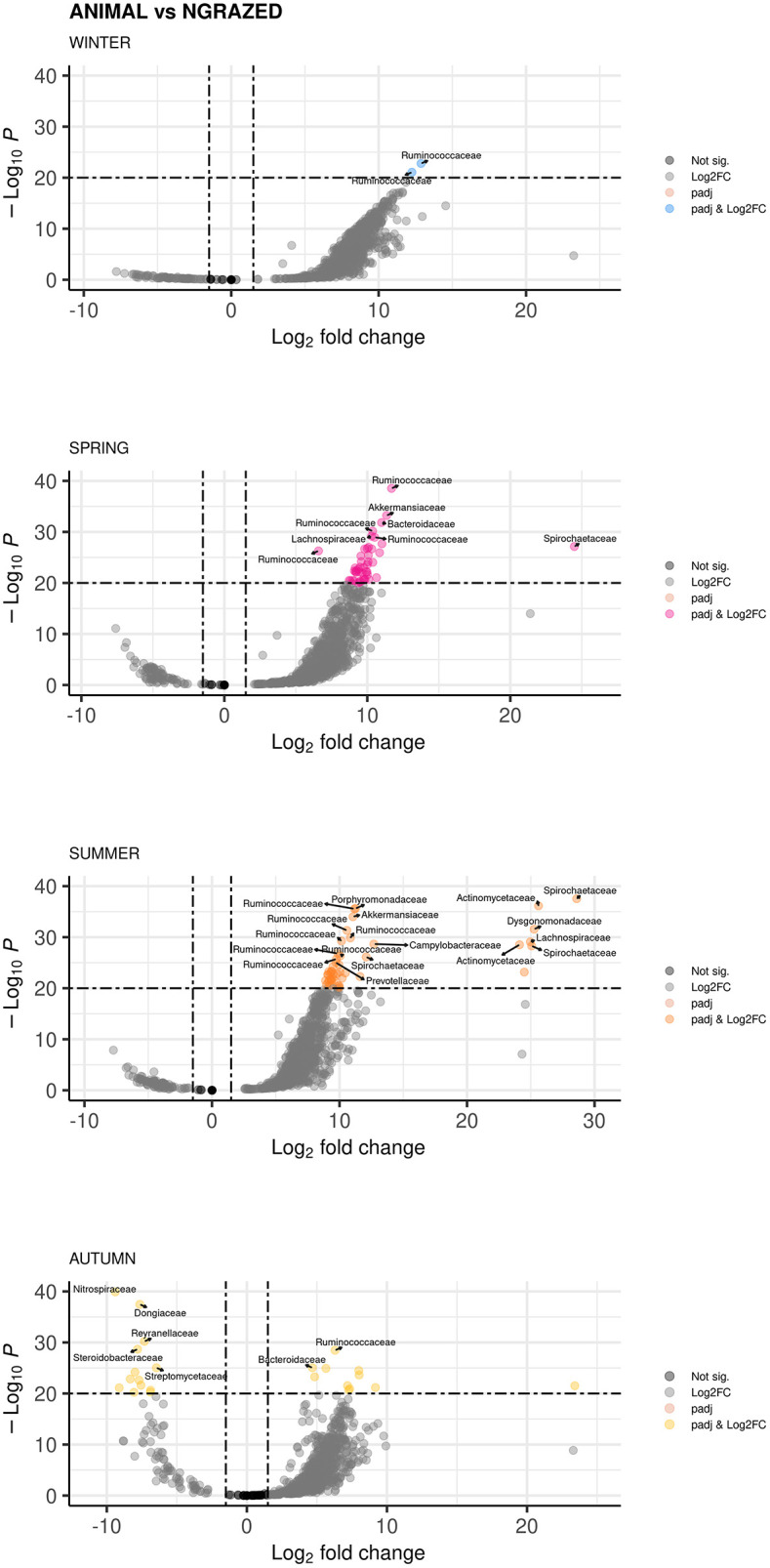
Volcano plots showing significant differences found at the Family level between ANIMAL and NGRAZED.

**Fig 8:**
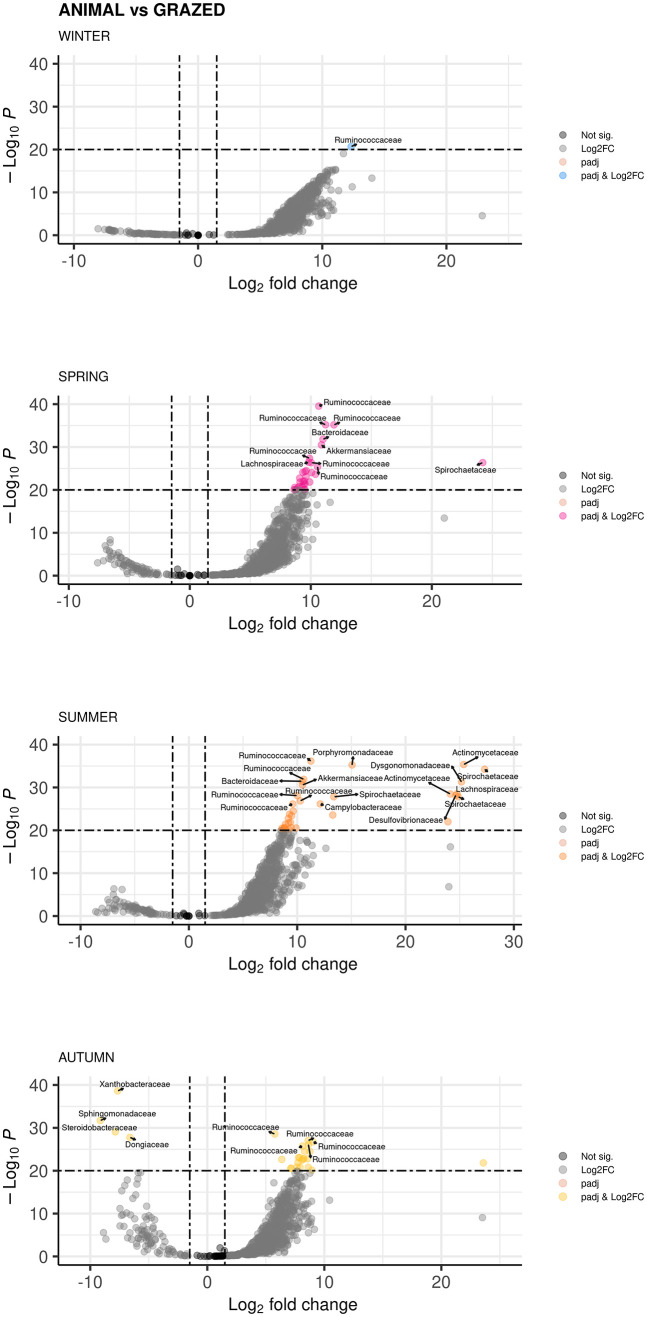
Volcano plots showing significant differences found at the Family level between ANIMAL and GRAZED.

**Fig 9:**
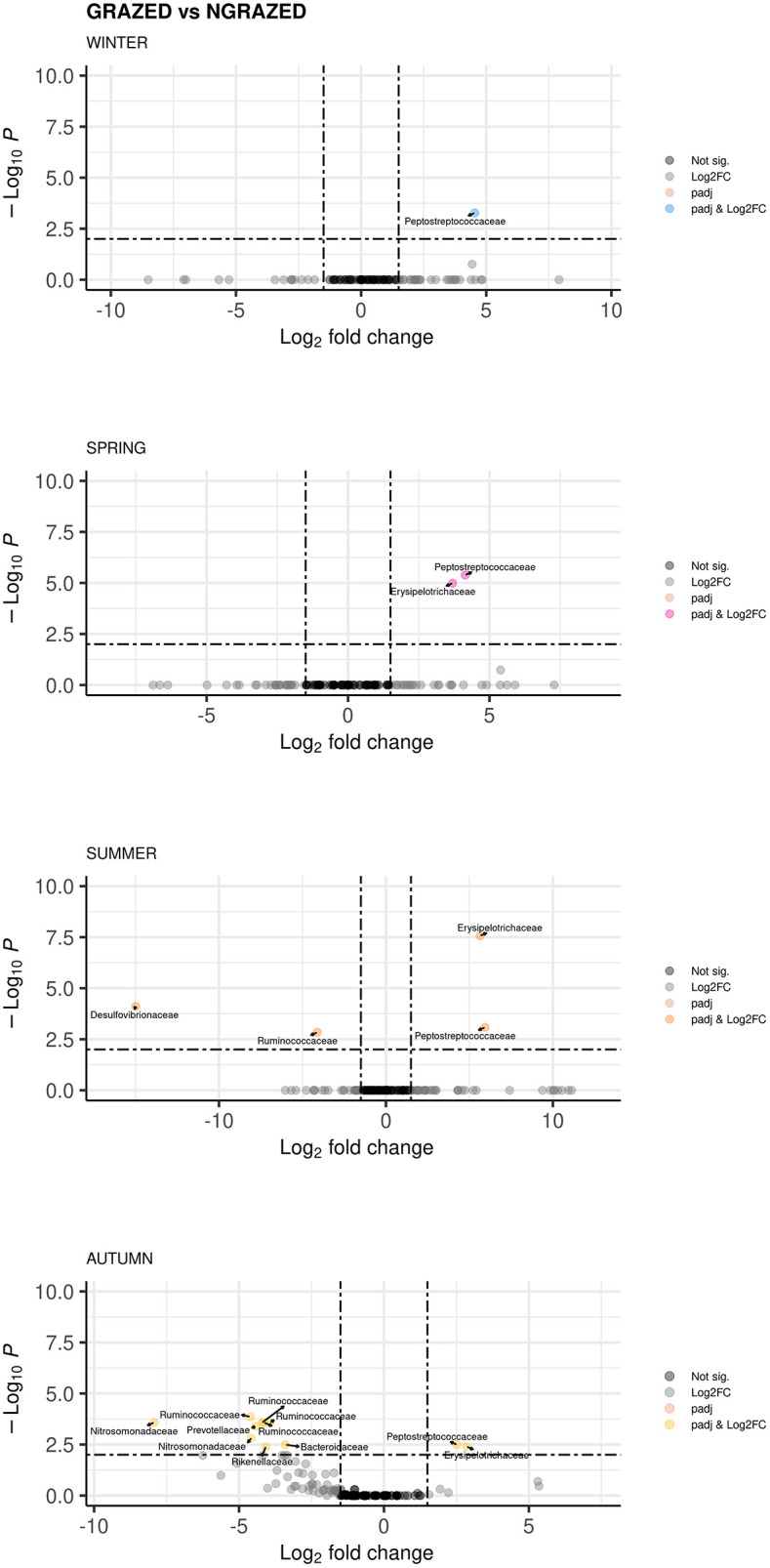
Volcano plots showing significant differences found at Family level between GRAZED and NGRAZED.

In this study, statistically significant differences in microbial abundance were observed among ANIMAL and soil samples. All pairwise comparisons — ANIMAL vs NGRAZED (Fig 7), ANIMAL vs GRAZED (Fig 8), GRAZED vs NGRAZED (Fig 9) — have been carried out for each season (details in S4 Table). It was considered as significant ASVs (in figures reported at the Family level) with a p-value adjusted ≤ 1e-20, and only the most significant non-overlapping ASVs have been annotated, to make the figures more comprehensible. Regarding soil comparisons, given the lower number of samples, a different threshold was applied (p-value adjusted ≤ 0.01, Fig 9), considering the consulted bibliography [23,24]. In total, 1029 ASVs were considered in the differential abundance analysis.

ANIMAL bacterial composition differed from that of NGRAZED during all seasons (Fig 7). Moving from Winter to Autumn, the number of ASVs significantly increased between the groups: in Winter, only the *Ruminococcaceae* family made the difference, being significantly more abundant in ANIMAL.

In Spring, *Lachnospiraceae*, *Bacteroidaceae*, *Akkermansiaceae*, and *Spirochaetaceae* increased in ANIMAL, as families able to discriminate between the two groups of samples. In Summer, *Actinomycetaceae*, *Porphyromonadaceae*, *Prevotellaceae*, *Campylobacteraceae,* and *Dysgonomonadaceae* families also increased and were significantly more abundant in the animal group. Interestingly, in Autumn, in NGRAZED soil samples, *Nitrospiraceae*, *Dongiaceae*, *Reyranellaceae*, *Steroidobasteraceae,* and *Streptomycetaceae* significantly increased, while only *Ruminococcaceae* and *Bacteroidaceae* remained significantly different in animals.

Again, ANIMAL differed from GRAZED during all seasons (Fig 8) and, from Winter to Autumn, the number of different ASVs significantly increased between the groups. In Winter, the *Ruminococcaceae* family was significantly more abundant in ANIMAL. In Spring, *Lachnospiraceae*, *Akkermansiaceae, Bacteroidaceae, and Spirochaetaceae* significantly distinguished animals from grazed samples.

In Summer, *Campylobacteraceae, Bacteroidaceae, Akkermansiaceae, Porphyromonadaceae, Spirochaetaceae, Dysgonomonadaceae, Actinomycetaceae,* and *Lachnospiraceae* families became detectable and significantly more abundant in ANIMAL. In Autumn, *Xanthobactriaceae*, *Sphingomanadaceae*, *Steroidobacteraceae* and *Dongiaceae* distinguished GRAZED from ANIMAL, because more abundant, while remaining significantly different the *Ruminococcaceae* in ANIMAL.

Also, GRAZED bacterial composition differed from NGRAZED during all seasons (Fig 9), and from Spring to Autumn, the number of different ASVs among groups increased. In Winter, the *Peptostreptococcaceae* family significantly increased in GRAZED soil. In Spring, the *Erysipelotrichaceae* also became more abundant and able to distinguish grazing soil from NGRAZED. In Summer, high levels of *Ruminococcaceae* and *Desulfovibrionceae* allowed for the characterization of the NGRAZED. In Autumn, *Nitrosomonadaceae*, *Prevotellaceae*, *Bacteroidaceae*, *Rikenellaceae*, and *Ruminococcaceae* families seemed to decrease their abundances in GRAZED, while *Peptostreptococcaceae* and *Erysipelotrichaceae* remained significantly high.

## Discussion

To the best of our knowledge, this study represents one of the first repeated cross-sectional, seasonal analyses of sheep and soil microbial interactivity.

The soils of the investigated areas showed relatively low bulk density (BD) values and quite good SOM content, suggesting a well-developed and stable structure. Even in the GRAZED area, where slight compaction was observed due to animal trampling, the overall BD values were quite good. The persistent grass cover plays a key role in forming and maintaining a porous, well-aggregated structure that facilitates water infiltration, root development, and biological activity. The continuous input of organic residues from plant litter and roots, together with soil fauna–driven bioturbation (e.g., by earthworms and microarthropods), supports the maintenance of low bulk density in surface horizons and mitigates the effects of mechanical compaction [25,26].

This preliminary assessment of physical and chemical soil properties aimed to account for potential confounding effects associated with inherent soil differences. Overall, the results indicated comparable properties between grazed and non-grazed areas, thereby supporting the interpretation of the subsequent microbial analyses and suggesting that the observed patterns were unlikely to be primarily driven by baseline soil variability.

### Taxonomic composition of animal and soil microbiomes

Existing literature shows that the relationship between grazing disturbance and microbial diversity is complex and not fully understood. Given the limited studies on sheep grazing systems, our findings add to the current knowledge and may help guide future research on soil microbial responses to grazing.

*Firmicutes, Proteobacteria, Bacteroidetes, Acidobacteria and Proteobacteria*, were the dominant bacteria in our study, though their relative abundances, where present, varied across the different samples. One bacterial phylum, *Actinobacteria*, was common to ANIMAL and soil samples, while six bacterial phyla, *Firmicutes, Proteobacteria, Bacteroidetes, Acidobacteria, Verrucomicrobia, and Cyanobacteria,* were differently distributed among the samples. Moreover, the phyla *Spirochaetes* and *Epsilonbacteriota* were present only in ANIMAL, while *Acidobacteria*, *Verrucomicrobia* and *Cyanobacteria* were detected only in soil samples.

On average, *Firmicutes, Bacteroides,* and *Spirochaetes* were predominant in all ANIMAL samples, as observed in literature [27]. A healthy and balanced microbiota is marked by the predominance of strict anaerobic species belonging to the phyla *Bacteroidetes* and *Firmicutes*. Members of *Bacteroidetes* possess a relatively high number of genes coding for carbohydrate-active enzymes, thus facilitating the degradation of structural polysaccharides in the rumen. Additionally, they are capable of fermenting amino acids to produce acetate. *Firmicutes*, on the other hand, the other most abundant bacterial group, include a wide range of fibrolytic and cellulolytic genera [28]. *Spirochaetes,* an important lignocellulose-degrading bacterial group, increased in Summer, when animals typically graze on lower-quality pasture. Notably, *Epsilonbacteraeota w*as detected only in Summer; this phylum has been negatively correlated with dry matter intake, which is known to decrease under heat stress [29]*.*

In GRAZED and NGRAZED soils, *Proteobacteria*, *Acidobacteria,* and *Actinobacteria* prevailed, which is in accordance with other studies on the soil bacteriome [30]. Bacteria belonging to these three phyla have been shown to be capable of degrading soil organic matter, thanks to the production of enzymes breaking down litter and dead wood into simpler molecules [31,32]. At the level of these three phyla, there were no significant differences between GRAZED and NGRAZED, except for an increase in *Acidobacteria* in spring and *Actinobacteria* in summer. Although grazing can contribute to increased nutrient inputs through the deposition of manure and urine, which in turn stimulates soil bacterial growth [33], this pattern was not evident in our study. Instead, the changes observed during the warmer seasons may have been more strongly influenced by increasing temperature.

Interestingly, the phylum *Actinobacteria* was present in all samples, including the ANIMAL samples. It is well known that the Gram-positive *Actinobacteria* play various important roles in soil beyond decomposing organic matter. For example, they are important in bioremediation and bioweathering, producing a wide range of bioactive compounds, and some genera of this phylum have recently been shown to be molecular biomarkers of landforms belonging to Mediterranean environments [34]. *Actinobacteria* are also present, albeit infrequently, in the rumen microbiota [35] where they also produce carbohydrate-active enzymes capable of degrading plant biomass during the animal’s digestion process [36]. Therefore, it is reasonable to have detected *Actinobacteria* in both the ANIMAL and soil samples. However, it is not possible to determine whether the *Actinobacteria* present in ANIMAL samples originate from soil ingestion or, conversely, were transferred from animals to the soil. Nonetheless, this result may encourage further studies to isolate *Actinobacteria* and the enzymes involved in plant cell wall degradation.

*Cyanobacteria* were present in greater quantities in GRAZED soil in Winter, decreasing over the subsequent seasons. Therefore, grazing introduced a disturbance, also leading to a detectable change in soil bacterial diversity, as *Bacteroidetes* and *Verrucomicrobia* became detectable (although in smaller quantities) between Spring and Summer. A possible explanation may be related to the more compacted conditions of the GRAZED soil, which could have influenced the presence of *Cyanobacteria.* Furthermore, animal excrement provides carbon sources for other heterotrophic microorganisms, favoring the introduction and survival of fecal bacteria (e.g., *Bacteroidetes*) in soil. Overall, in the soils analyzed, grazing can negatively affect the abundance of dominant groups such as *Cyanobacteria*. This hypothesis is supported by other studies that specifically investigated the effects of grazing on cyanobacterial community composition [37,38].

At the family level, a great microbial diversity was evident from Winter to Autumn, both in the ANIMAL and soil samples. The five most abundant families in the ANIMAL group were *Ruminococcaceae*, *Spirochaetaceae*, *Porphyromonadaceae*, *Lachnospiraceae*, and *Rikenellaceae*, in line with findings from other studies [29,39]. Based on our results and literature concerning different sheep breeds [39,40], *Lachnospiraceae* and *Rikenellaceae* could potentially be used as biomarkers for heat stress, decreasing during Summer. These families are generally linked to animal health. *Lachnospiraceae* produce short-chain fatty acids and inhibit intestinal pathogens, and have been associated with milk production; some studies report a negative correlation with yield [41,42], while others find a positive correlation [43,44]. Therefore, while they likely influence milk production, the exact mechanisms remain unclear. *Rikenellaceae* are important for maintaining mucosal immunity and enhancing metabolism [45]. The increase in *Porphyromonadaceae* detected here was also reported in beef cattle by Liu et al. (2020) [46], and was a discriminative feature separating grass-fed animals from the grain-fed group. This suggests that this Family increases when animals graze. The last interesting aspect identified here was the increase in the *Spirochaetaceae family* in hot periods. Specifically, it is the genus *Tremonema_2* that increases in Summer (Fig 4). Several treponema taxa are associated with human and cattle diseases, but lastly, several treponema have also been reported to be commensal, symbionts in the gastrointestinal tracts of animals [47]. The reason why it decreased in hot periods from Summer to Autumn could be related to animal heat stress.

In soil samples, one of the most notable findings was the absence of bacterial families typically found in ANIMAL samples across both GRAZED and NGRAZED soils. Instead of a transfer of fecal bacterial families, a progressive increase in the number of soil bacterial families was observed from Winter to Autumn. However, since this increase occurred in both GRAZED and NGRAZED soils, it is more likely to be associated with seasonal changes — likely particularly rising environmental temperatures — than with the direct influence of sheep grazing. Although the fecal bacteriome also exhibited greater biodiversity in the warmer seasons, there was no clear evidence of its bacterial families establishing in GRAZED soils. This suggests that temperature-driven changes in soil microbial composition may play a more significant role than grazing pressure. Similar observations have been reported in previous studies, where warming alone led to shifts in bacterial communities [48,49].

Four families are the most represented in the two soil samples in Winter: *Sphingomonadaceae*, *Xanthobacteriaceae*, *Chthoniobacteraceae*, and *Bacillaceae*. Of these, *Sphingomonadaceae* and *Xanthobacteriaceae* remain detectable even in the other seasons, albeit with varying abundances, while *Chthoniobacteraceae* and *Bacillaceae* decrease or remain below the detection threshold as the seasons progress. *Sphingomonadaceae* and *Xanthobacteriaceae* are ubiquitous in soils and frequently found in polluted environments and in the formation of membrane biofilms [50]; but they are also known to be N fixers in soil and contain many species that can reduce sulfur and hydrogen compounds [51]. Therefore, this result is not particularly surprising. *Chthoniobacteraceae* (phylum: *Verrucomicrobia*) are associated with the C cycle in soil; positively correlated with Soil Organic Carbon and soil N levels, and negatively correlated with the pH level [52].

*Bacillaceae* are distributed in many natural environments, including sediments, water, air, marine ecosystems, activated sludge, and human and animal systems, as well as soil. Their most notable characteristic is their ability to form endospores, enabling them to withstand many environmental stresses for a long time [53]. We detected this family only in Winter soils and, to a lesser extent, in GRAZED soils in Summer. Although the reasons for this pattern remain unclear, it is possible that seasonal conditions in the other sampling periods were less favorable to the growth of *Bacillaceae*, reducing their relative abundance below the detection threshold during the study period.

At the genus level, Ruminococcaceae_UCG.005 and Ruminococcaceae_UCG.010 were the two most abundant genera due to their well-known role in the degradation of cellulose and starch. The former was more abundant than the latter, as was described by several authors [54,55]. Unfortunately, no studies have been found comparing directly the *Ruminococcaceae_UCG.005* and the *Ruminococcaceae_UCG.010* across the same seasonal framework considered here, although seasonal fluctuations of *Ruminococcaceae* have been previously described [40]*.*

Regarding the soil communities’ relative abundance, the results mirror those observed at the family level. Furthermore, the *Sphingomonas* genus persists throughout the seasons. Indeed, this bacterial genus is known for its ability to degrade lignin, phenolic acid, and physiological characteristics that allow it to adapt to many environments [56].

The *Bacillus* genus, on the other hand, is highly present in GRAZED and NGRAZED soils in Winter, while, in the other seasons it was detected only in GRAZED soils and at lower abundance, consistent with the pattern observed at the Family level. This may suggest a strong influence of seasonal conditions, including temperature, on the abundance of this bacterial genus, together with other climate-related factors affecting soil *Bacillus* population [57].

### Alpha and beta diversity indices

In our study, we observed a significant difference in the Chao1 richness index across sample types (p < 0.0001), with notably higher values in the animal-associated microbiota compared to soil. In contrast, the Shannon diversity index did not reveal statistically significant differences between these groups. This apparent discrepancy arises from the different aspects of diversity each metric captures. The Chao1 index estimates total species richness, emphasizing unobserved or rare taxa, which are more prevalent in the animal microbiome due to its greater variability. In contrast, Shannon’s index considers both richness and evenness and is less sensitive to rare taxa, reflecting the balance of species abundances. In communities where a few taxa dominate, Shannon values may be similar despite differences in total richness [58]. Soil communities tend to be more evenly distributed and show fewer rare taxa, resulting in similar Shannon diversity but lower Chao1 richness compared to animal samples. This pattern highlights the importance of using multiple alpha-diversity indices to capture different ecological facets of microbial diversity. Alpha diversity values align with those reported in the literature for fecal samples [59]. The Shannon index showed no significant differences among groups or seasons, indicating that observed diversity differences are primarily due to species richness rather than evenness.

Beta diversity analysis revealed a strong group effect observed in all seasons. The within-group variability in ANIMAL samples, as shown by PCoA, was higher than expected, especially considering the inclusion of soil samples, which typically increase between-group differences and reduce within-group variability. Although no comparable studies were found, individual animal effects — such as physiological stage and health status — likely contribute significantly, as both these factors are known to influence gut microbiota [60].

PERMANOVA results indicated that the group factor explained a significant portion of the variation in microbial communities across all seasons (R² from 0.32 to 0.44, p < 0.0001), highlighting a consistent and prominent influence of host (sheep vs. soil) and environment (grazed vs. ungrazed) throughout the study period. In other words, analyses of alpha and beta diversity indicated that the dominant source of variation was the type of sample (feces versus soil) rather than grazing management. Fecal microbiomes displayed higher bacterial richness and different composition than both grazed and ungrazed soils, with minor seasonal fluctuations. These patterns align with previous studies reporting that livestock fecal microbiota is compositionally distinct when compared to soils [61,62]. Seasonal changes of the sheep gut microbiome are well recognized and vary according to environment, diet, and management practices, supporting the moderate seasonal shifts observed here [40]. However, it is important to note that significant differences in multivariate dispersion were detected in Spring and Summer. This suggests that, during these seasons, part of the variation captured by PERMANOVA may be influenced by differences in within-group variability. Such heterogeneity in dispersion may reflect increased environmental variability and biological heterogeneity during warmer periods, consistent with the dynamic nature of microbiome responses to temperature and grazing activity. In contrast, differences between grazed and ungrazed soils were weak, a result consistent with several findings in the literature: heavy grazing can reduce soil bacterial diversity through pH and nutrient alterations [63], whereas light or moderate grazing and grazing exclusion often lead to neutral or increased diversity [64,65].

### Differential abundance analysis

Identifying significant differentially abundant features in this study provides insights into the influence of animal fecal inoculum on soil and allows comparison of soil quality and structure post-grazing. Analyzing data seasonally helps identify potential biomarkers linked to specific conditions, such as heat stress or ecological processes. Across all comparisons, differentially abundant ASVs corresponded to families typically dominant in each group. In particular, *Ruminococcaceae* consistently differed between ANIMAL and both GRAZED and NGRAZED across seasons. From Spring to Autumn, the number of differentially abundant ASVs increased, including families such as *Lachnospiraceae*, *Spirochaetaceae*, and *Porphiromonadaceae* — the most abundant in animal feces — as well as others like *Akkermansiaceae*, *Actinomycetaceae*, *Campylobacteraceae*, and *Dysgonomonadaceae*.

These families appeared in both soil comparisons, ANIMAL vs NGRAZED (Fig 7) and ANIMAL vs GRAZED (Fig 8). The Family *Akkermansiaceae* is found to play a key role in regulating metabolic health and controlling inflammatory status [66]. The presence in hotter months could be related to both the diet change and the necessity to modulate the immune response. The *Actinomycetaceae* were found to be significantly different from Spring to Autumn [67] between animal and soil. The increase in *Actinomycetaceae* during the grazing period likely reflects their role in lignocellulosic feed digestion [68]. Notably, Klitgaard et al. (2017) [69] identified *Actinomycetaceae* associated with digital dermatitis in dairy cattle, and here, the genus Treponema was also found to increase in hotter seasons (Fig 4) suggesting that it might be linked to seasonal effects. Further research is needed to assess whether these microorganisms could serve as biomarkers of general animal discomfort, as no health issues were reported in the animals during the study. Additionally, *Campylobacteraceae* appears to be seasonally influenced in ruminants, explaining its differential abundance in warmer sampling periods [70]. To date, no studies have explored the seasonal fluctuations of *Dysgonomonadaceae*.

Findings of differentially abundant ASVs between grazed and ungrazed soil (Fig 9) could support the hypothesis of fecal microbiota being transferred to the soil, but further studies using direct tracking methods would be needed to confirm it. During Winter, Spring, and Summer, grazed soil showed distinct levels of *Peptostreptococcaceae* and *Erysipelotrichaceae*. Although *Peptostreptococcaceae* is prominent in dairy cattle [3,71], its presence in sheep was minimal but still effectively distinguished grazed from non-grazed soils. The presence of *Erysipelotrichaceae* in grazed soil is particularly noteworthy as, in dairy cows, this family is linked to inflammation, infectious diseases, ruminal acidosis, and responds to dietary changes [72,73]. Higher levels in thin cows suggest that *Erysipelotrichaceae* could serve as a health biomarker, potentially applicable to sheep as well.

In Summer and Autumn, *Ruminococcaceae* also differed between soil types; its occurrence in soil aligns with previous findings of this family’s presence in anaerobic soil hotspots [74].

Overall, the differential abundance analysis highlighted *Peptostreptococcaceae* and *Erysipelotrichaceae* for all seasons; *Ruminococcaceae* and *Desulfovibrionaceae* in Summer; and *Nitrosomonadaceae*, *Prevotellaceae*, *Bacteroidaceae*, and *Rikenellaceae* in Autumn. These bacterial families could be considered indicators of a change in community composition and as discriminant factors among groups. Thus, what could not be highlighted by Relative Abundance Analysis was demonstrated by Differential Abundance Analysis.

## Conclusions

This study represents one of the first analyses examining the seasonal dynamics of the sheep fecal microbiome and the influence of grazing on soil bacterial communities in an extensive farming system. Results revealed clear differentiation between animal fecal and soil microbiota, while also showing that grazing significantly alters soil bacterial composition. Notably, microbial families such as *Peptostreptococcaceae* and *Erysipelotrichaceae* were more abundant in grazed soils, suggesting potential microbial transfer from animals to the environment. Within the fecal microbiome, seasonal variations highlighted bacterial families associated with heat stress, including decreases in *Lachnospiraceae* and *Rikenellaceae* and increases in *Spirochaetaceae* and *Porphyromonadaceae* during summer. These changes indicate potential biomarkers for monitoring animal physiological stress under heat conditions. However, this study has some limitations that should be considered when interpreting the findings. Indeed, sampling was restricted to lactating ewes, and soil sampling was limited to bulk soil collected at a single depth (10 cm), without comparison across soil compartments (e.g., bulk vs. rhizosphere) or depth/soil horizons. In addition, no measures of vegetation structure or plant-associated microbiota were included.

Overall, this investigation underscores the importance of seasonally stratified (repeated cross-sectional) studies to understand animal-soil microbiome interactions and their implications for sustainable grazing management, animal health, and soil ecosystem function in agro-pastoral systems.

## Supporting information

S1 Fig PCoA zoomed on soil samples. (PDF)

S1 Table Main physico-chemical characteristics of the soil samples collected in the GRAZED and NGRAZED areas. (PDF)

S2 Table ASV-level relative abundances for sample and the corresponding alpha diversity indices. (CSV)

S3 Table Pairwise group-comparisons of PERMANOVA. (PDF)

S4 Table Lists od significant taxa obtained in Differential Abundance Analysis. (PDF)
