# Supplementary figures and images for: Seasonal dynamics in sheep fecal microbiome and soil bacterial communities under grazing management
Source: PLoS One. 2026 Jun 29;21(6):e0352436. doi: 10.1371/journal.pone.0352436 (PMC13313378; doi:10.1371/journal.pone.0352436)

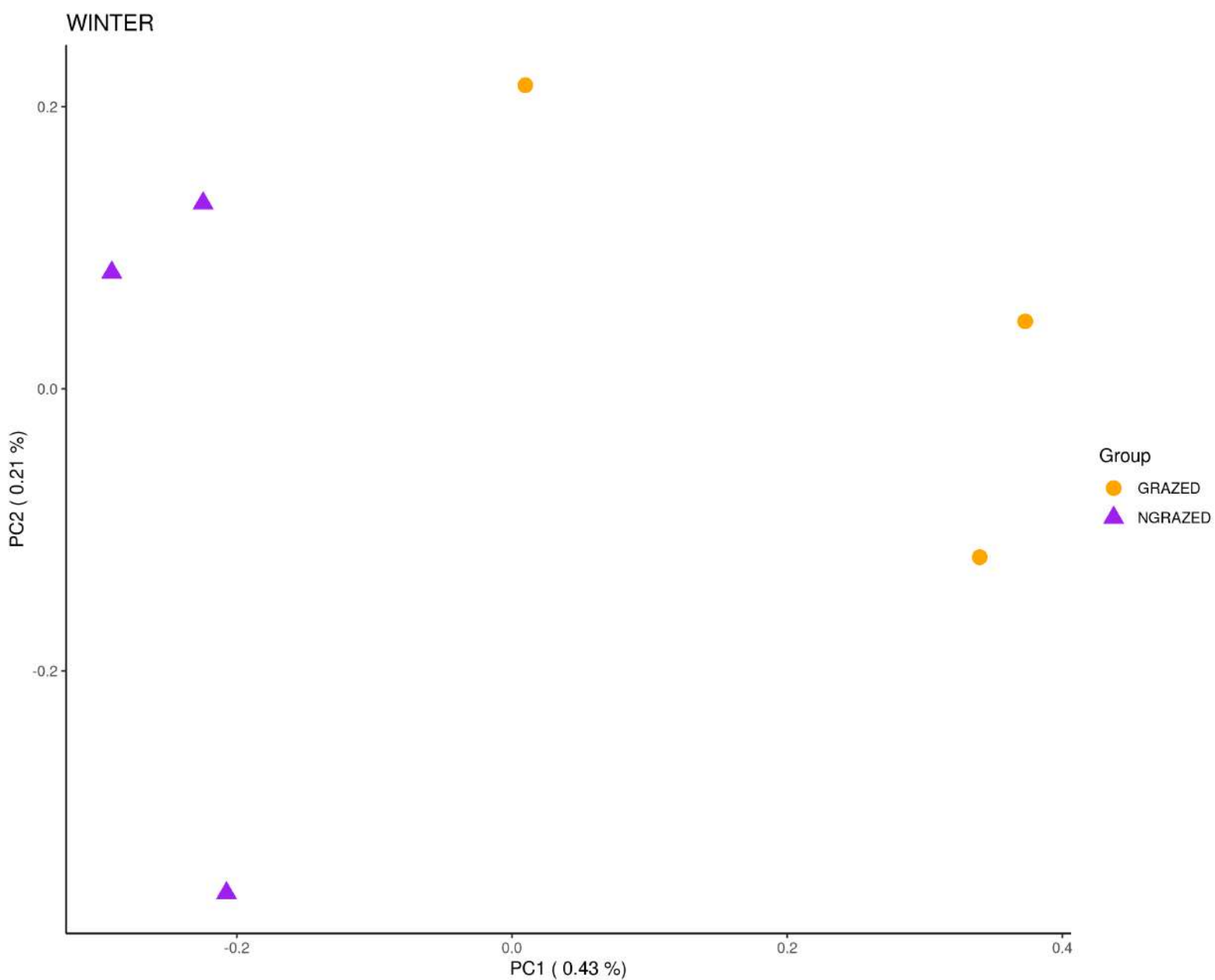

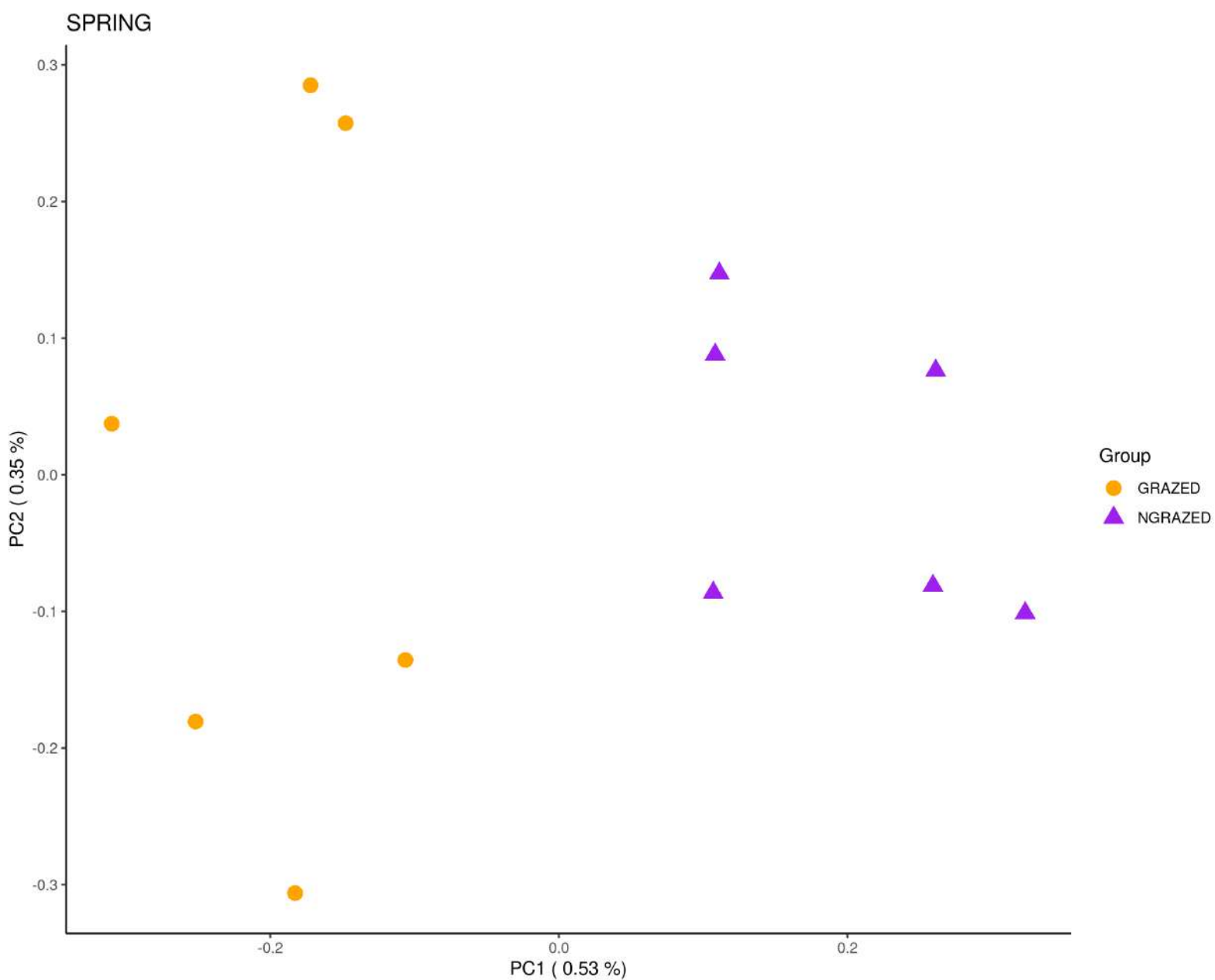

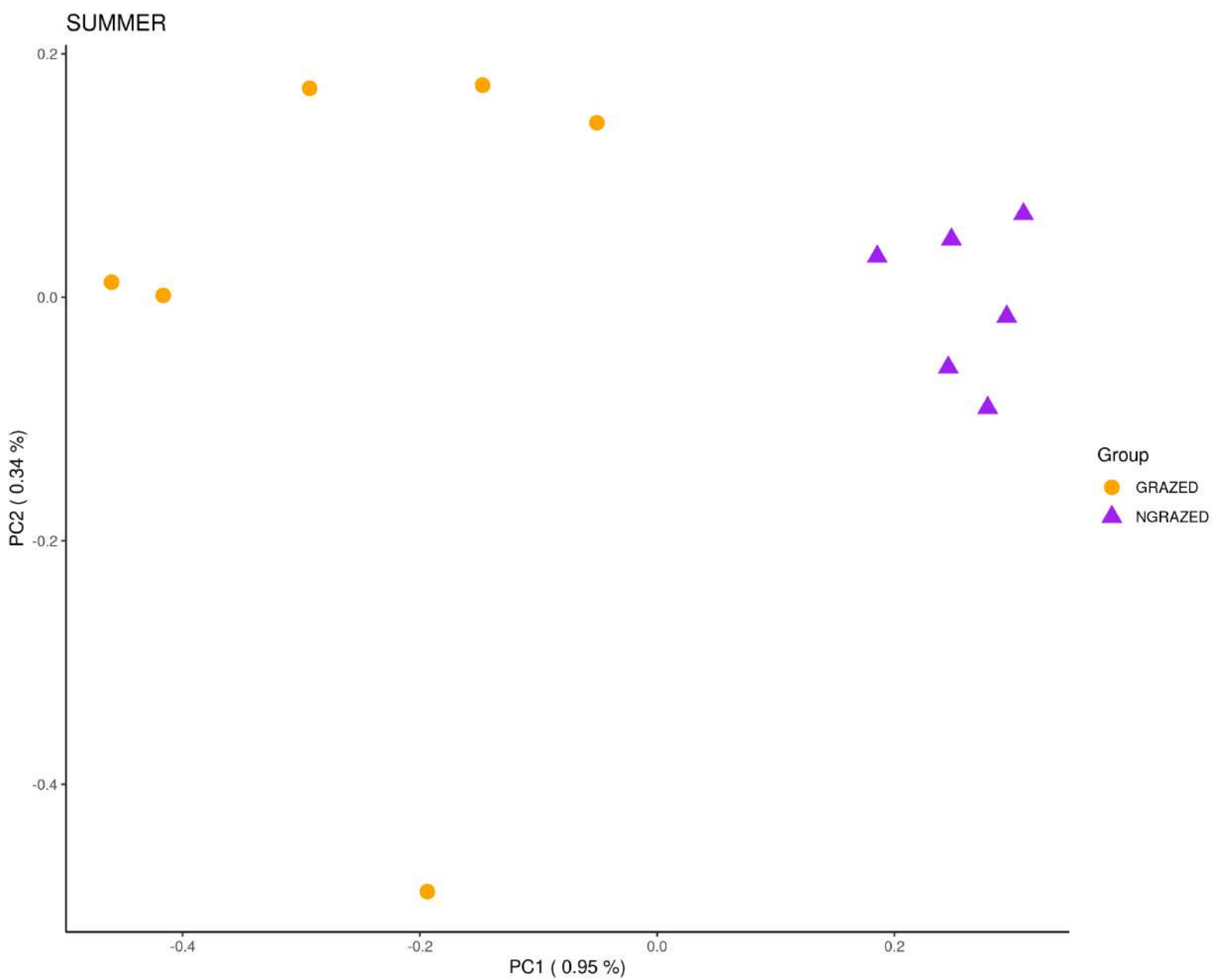

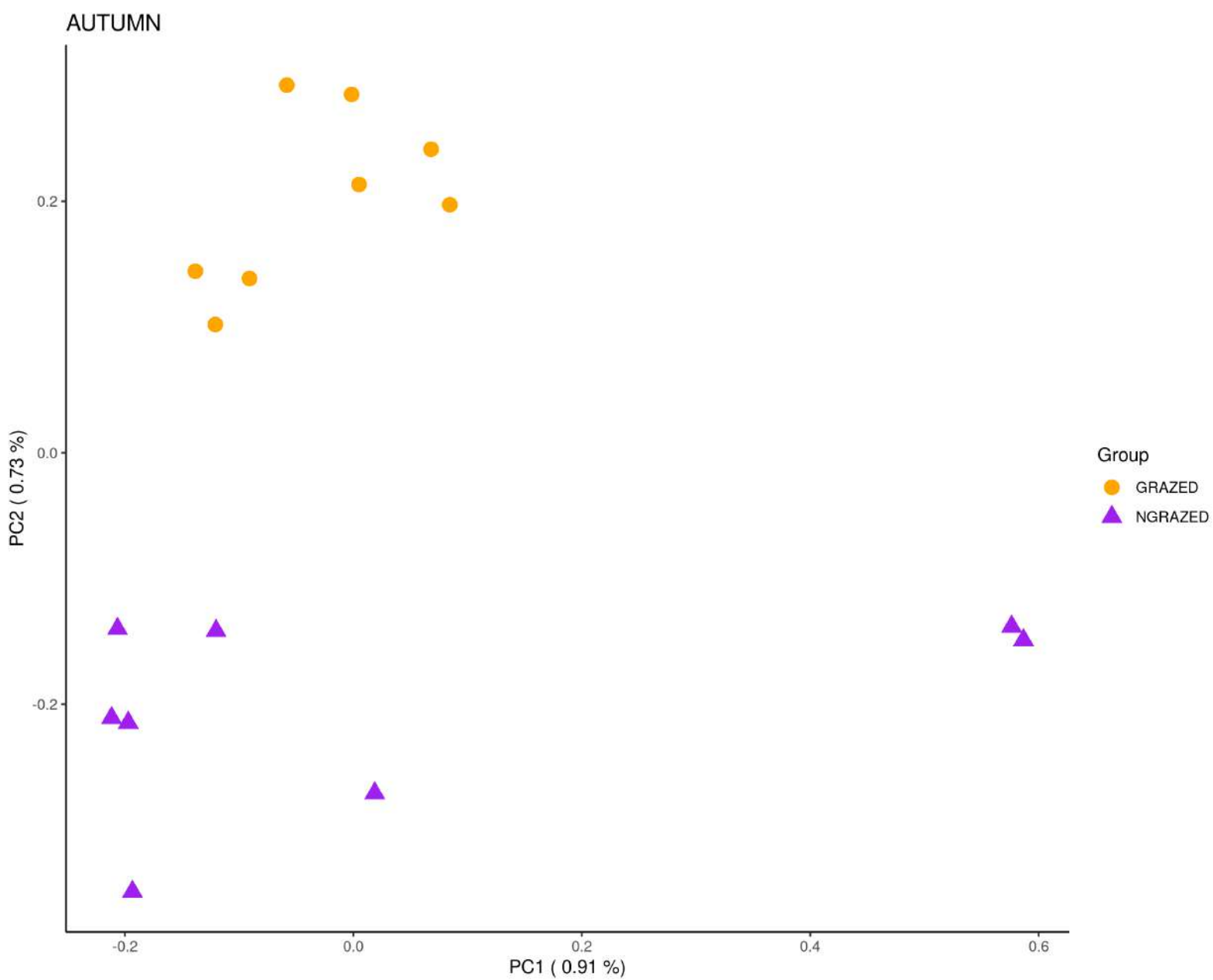

Supplement: S1 Fig — (PDF) [file pone.0352436.s001.pdf]
