# Supplementary material for: Seasonal dynamics in sheep fecal microbiome and soil bacterial communities under grazing management
Source: PLoS One. 2026 Jun 29;21(6):e0352436. doi: 10.1371/journal.pone.0352436 (PMC13313378; doi:10.1371/journal.pone.0352436)
Supplement: S1 Table — (PDF) [file pone.0352436.s002.pdf]

	Bulk Density (g/cm^3)	pH	EC (μS/cm)	Soil Organic Matter (%)
GRAZED	1.22	6.8	1.78	5.84
	1.17	6.7	1.6	5.34
	1.35	6.7	2.01	5.69
	1.28	6.9	1.43	5.85
	1.33	6.8	1.59	4.89
NGRAZED	1.04	6.8	1.83	5.39
	0.94	6.7	2.13	5.40
	1.13	6.7	1.45	5.40
	1.28	6.6	1.47	4.89
	1.07	7.2	1.79	5.59
