# Supplementary material for: Seasonal dynamics in sheep fecal microbiome and soil bacterial communities under grazing management
Source: PLoS One. 2026 Jun 29;21(6):e0352436. doi: 10.1371/journal.pone.0352436 (PMC13313378; doi:10.1371/journal.pone.0352436)
Supplement: S3 Table — (PDF) [file pone.0352436.s004.pdf]

		Df	SumOfSqs	R2	F	Pvalue
				ANIMAL_vs_GRAZED		
**WINTER**	Model	1	2 .12	0 .22	14 .24	0 .0002
	Residual	51	7 .58	0 .78		
	Total	52	9 .69	1		
				ANIMAL_vs_NGRAZED		
	Model	1	2 .13	0 .22	14 .39	0 .0001
	Residual	51	7 .55	0 .78		
	Total	52	9 .69	1		
				GRAZED_vs_NGRAZED		
	Model	1	0 .37	0 .42	2 .86	0 .1
	Residual	4	0 .52	0 .58		
	Total	5	0 .89	1		
				GRAZED_vs_NGRAZED		
**SPRING**	Model	1	0 .46	0 .31	4 .56	0 .0035
	Residual	10	1 .02	0 .69		
	Total	11	1 .48	1		
				GRAZED_vs_ANIMAL		
	Model	1	3 .95	0 .31	25 .7	0 .0001
	Residual	58	8 .91	0 .69		
	Total	59	12 .87	1		
				NGRAZED_vs_ANIMAL		
	Model	1	4	0 .31	26 .16	0 .0001
	Residual	58	8 .86	0 .69		
	Total	59	12 .86	1		
				GRAZED_vs_ANIMAL		
**SUMMER**	Model	1	3 .61	0 .2	18 .36	0 .0001
	Residual	74	14 .53	0 .8		
	Total	75	18 .14	1		
				GRAZED_vs_NGRAZED		
	Model	1	0 .83	0 .44	7 .72	0 .0032
	Residual	10	1 .08	0 .56		
	Total	11	1 .91	1		
				ANIMAL_vs_NGRAZED		
	Model	1	4 .04	0 .22	21 .29	0 .0001
	Residual	74	14 .05	0 .78		
	Total	75	18 .1	1		
				GRAZED_vs_NGRAZED		
**AUTUMN**	Model	1	0 .66	0 .21	3 .63	0 .0003
	Residual	14	2 .55	0 .79		
	Total	15	3 .21	1		
				GRAZED_vs_ANIMAL		
	Model	1	4 .33	0 .36	25 .05	0 .0001
	Residual	44	7 .6	0 .64		
	Total	45	11 .92	1		
				NGRAZED_vs_ANIMAL		
	Model	1	3 .82	0 .32	20 .79	0 .0001
	Residual	44	8 .08	0 .68		

Total	45	11 .9	1
