# Supplementary material for: Seasonal dynamics in sheep fecal microbiome and soil bacterial communities under grazing management
Source: PLoS One. 2026 Jun 29;21(6):e0352436. doi: 10.1371/journal.pone.0352436 (PMC13313378; doi:10.1371/journal.pone.0352436)
Supplement: S4 Table — (PDF) [file pone.0352436.s005.pdf]

Comparisons	Season	N	taxon	baseMean	log2FoldChange	lfcSE	stat	pvalue	padj	Family	Genus
GRAZED vs ANIMAL	WIN	1	ASV_5	218,6043	12,32487	1,20459	10,23159	1,43E-24	2,08E-21	Ruminococcaceae	Ruminococcaceae_UCG-005
	SPR	1	ASV_11	231,3702	11,90903	0,916713	12,99101	1,38E-38	6,71E-36	Ruminococcaceae	Ruminococcaceae_UCG-005
		2	ASV_112	33,14075	9,107399	0,896188	10,16237	2,92E-24	1,47E-22	Christensenellaceae	Christensenellaceae_R-7_group
		3	ASV_115	46,84019	9,605111	0,918637	10,45583	1,38E-25	9,16E-24	Ruminococcaceae	Ruminococcaceae_UCG-005
		4	ASV_12	143,9171	11,22449	0,86169	13,02614	8,69E-39	6,35E-36	Ruminococcaceae	Ruminococcaceae_UCG-005
		5	ASV_124	43,90001	9,511019	0,978413	9,720859	2,46E-22	8,62E-21	Acidaminococcaceae	Phascolarctobacterium
		6	ASV_127	41,24008	9,419758	0,938002	10,04236	9,93E-24	4,54E-22	Lachnospiraceae	Lachnospiraceae_NK4A136_group
		7	ASV_128	32,62044	9,080552	0,87413	10,3881	2,81E-25	1,79E-23	Desulfovibrionaceae	Mailhella
		8	ASV_129	26,06868	8,760073	0,898554	9,74908	1,86E-22	7,16E-21	Ruminococcaceae	Ruminococcaceae_UCG-002
		9	ASV_133	37,1763	9,272033	0,935809	9,908042	3,84E-23	1,65E-21	Bacteroidaceae	Bacteroides
		10	ASV_14	123,883	11,00777	0,890508	12,36122	4,24E-35	1,55E-32	Bacteroidaceae	Bacteroides
		11	ASV_146	31,21747	9,018318	0,909621	9,914364	3,61E-23	1,6E-21	Ruminococcaceae	Ruminococcaceae_UCG-005
		12	ASV_15	113,1256	10,87756	0,899194	12,09702	1,1E-33	3,2E-31	Akkermansiaceae	Akkermansia
		13	ASV_166	29,14009	8,919598	0,908661	9,816203	9,59E-23	3,79E-21	Lachnospiraceae	Lachnospiraceae_NK4A136_group
		14	ASV_169	42,30247	9,457507	0,972985	9,720096	2,48E-22	8,62E-21	Ruminococcaceae	Ruminococcaceae_UCG-005
		15	ASV_173	23,6837	8,622529	0,88647	9,726817	2,32E-22	8,47E-21	Ruminococcaceae	Ruminococcaceae_UCG-005
		16	ASV_21	90,47127	10,55541	0,960575	10,98865	4,33E-28	4,22E-26	Ruminococcaceae	Ruminococcaceae_UCG-005
		17	ASV_283	24,55542	8,675958	0,880016	9,858861	6,28E-23	2,55E-21	Ruminococcaceae	Ruminococcaceae_UCG-005
		18	ASV_29	44,43221	9,529778	0,9477	10,05569	8,67E-24	4,09E-22	Ruminococcaceae	Ruminococcaceae_UCG-002
		19	ASV_32	57,94222	9,911635	0,975091	10,16483	2,85E-24	1,47E-22	Prevotellaceae	Prevotellaceae_UCG-004
		20	ASV_320	37,60613	24,21125	2,154438	11,23785	2,66E-29	4,32E-27	Spirochaetaceae	Treponema_2
		21	ASV_36	63,37278	10,04108	0,894374	11,22693	3,01E-29	4,4E-27	Ruminococcaceae	Ruminococcaceae_UCG-005
		22	ASV_48	43,27662	9,490511	0,927626	10,23097	1,44E-24	8,43E-23	Ruminococcaceae	Ruminococcaceae_UCG-005
		23	ASV_5	344,1496	10,65915	0,77131	13,81954	1,94E-43	2,84E-40	Ruminococcaceae	Ruminococcaceae_UCG-005
		24	ASV_58	54,44697	9,82104	0,871737	11,26606	1,93E-29	3,53E-27	Lachnospiraceae	Lachnoclostridium_10
		25	ASV_60	64,5337	10,06702	0,941957	10,68736	1,17E-26	8,98E-25	Rikenellaceae	Alistipes
		26	ASV_63	57,43323	9,898945	0,862507	11,47695	1,72E-30	4,2E-28	Ruminococcaceae	Ruminococcaceae_UCG-005
		27	ASV_72	80,60156	10,38718	0,980271	10,59623	3,1E-26	2,27E-24	Spirochaetaceae	Treponema_2
		28	ASV_76	44,15921	9,521654	0,886384	10,74213	6,45E-27	5,55E-25	Ruminococcaceae	Ruminococcaceae_UCG-005
		29	ASV_78	38,98946	9,341074	0,872694	10,70372	9,78E-27	7,94E-25	Ruminococcaceae	Candidatus_Soleaferrea
		30	ASV_87	47,34118	9,62229	0,890876	10,80093	3,41E-27	3,11E-25	Ruminococcaceae	Ruminococcaceae_UCG-009

		31	ASV_95	55,0861	9,358081	0,916319	10,21269	1,74E-24	9,78E-23	Spirochaetaceae	Treponema_2
	SUM	1	ASV_1	1744,439	15,06463	1,162022	12,96416	1,95E-38	5,77E-36	Porphyromonadaceae	Porphyromonas
		2	ASV_102	29,41672	9,184007	0,885286	10,37405	3,25E-25	1,66E-23	Ruminococcaceae	Ruminococcaceae_UCG-010
		3	ASV_106	25,30549	8,956786	0,922059	9,713896	2,63E-22	8,1E-21	Ruminococcaceae	Ruminococcaceae_NK4A214_group
		4	ASV_11	63,13451	10,28263	0,911602	11,27974	1,65E-29	1,36E-27	Ruminococcaceae	Ruminococcaceae_UCG-005
		5	ASV_118	59,2546	24,62886	2,130912	11,5579	6,73E-31	7,1E-29	Desulfovibrionaceae	Desulfovibrio
		6	ASV_12	78,88384	10,60043	0,859487	12,33343	5,98E-35	1,26E-32	Ruminococcaceae	Ruminococcaceae_UCG-005
		7	ASV_128	22,6524	8,817178	0,894534	9,856726	6,41E-23	2,37E-21	Desulfovibrionaceae	Mailhella
		8	ASV_13	226,9293	12,12175	1,088974	11,13135	8,83E-29	6,86E-27	Campylobacteraceae	Campylobacter
		9	ASV_14	71,0437	10,45318	0,854174	12,23776	1,95E-34	3,61E-32	Bacteroidaceae	Bacteroides
		10	ASV_15	72,85442	10,48439	0,86977	12,0542	1,84E-33	2,72E-31	Akkermansiaceae	Akkermansia
		11	ASV_159	21,64342	8,734392	0,895136	9,75761	1,71E-22	5,5E-21	Ruminococcaceae	Ruminococcaceae_UCG-010
		12	ASV_219	46,30862	24,86356	2,167036	11,47353	1,79E-30	1,56E-28	Spirochaetaceae	Treponema_2
		13	ASV_221	40,07339	24,75747	2,12585	11,64592	2,41E-31	2,96E-29	Lachnospiraceae	Lachnoclostridium
		14	ASV_229	19,62128	8,591693	0,875505	9,813419	9,86E-23	3,31E-21	Ruminococcaceae	Ruminococcaceae_UCG-004
		15	ASV_26	48,23323	9,891776	1,006108	9,831724	8,22E-23	2,82E-21	Rikenellaceae	Rikenellaceae_RC9_gut_group
		16	ASV_29	52,25809	10,01067	0,867324	11,54202	8,1E-31	7,98E-29	Ruminococcaceae	Ruminococcaceae_UCG-002
		17	ASV_3	541,2238	13,37614	1,165147	11,48021	1,66E-30	1,53E-28	Spirochaetaceae	Treponema_2
		18	ASV_31	72,28912	25,15877	2,06182	12,20221	3,02E-34	4,96E-32	Dysgonomonadaceae	Proteiniphilum
		19	ASV_32	39,9208	9,625871	0,893844	10,76907	4,82E-27	3,23E-25	Prevotellaceae	Prevotellaceae_UCG-004
		20	ASV_35	29,76205	9,204715	0,908803	10,1284	4,13E-24	1,8E-22	Ruminococcaceae	Ruminococcaceae_UCG-010
		21	ASV_36	39,01877	9,58709	0,862018	11,12169	9,84E-29	7,27E-27	Ruminococcaceae	Ruminococcaceae_UCG-005
		22	ASV_368	25,42043	23,91357	2,34704	10,18882	2,22E-24	1,03E-22	Deferribacteraceae	Mucispirillum
		23	ASV_4	377,0439	27,30121	2,137367	12,77329	2,31E-37	5,69E-35	Spirochaetaceae	Treponema_2
		24	ASV_40	35,96432	9,464453	0,960631	9,852328	6,7E-23	2,41E-21	Acidaminococcaceae	Phascolarctobacterium
		25	ASV_44	36,18789	9,484988	0,901698	10,51904	7,06E-26	3,86E-24	Ruminococcaceae	Ruminococcaceae_UCG-009
		26	ASV_5	124,4975	11,25976	0,855449	13,16239	1,44E-39	7,11E-37	Ruminococcaceae	Ruminococcaceae_UCG-005
		27	ASV_52	130,9187	25,3691	1,951309	13,00107	1,21E-38	4,45E-36	Actinomycetaceae	Arcanobacterium
		28	ASV_55	115,519	24,1328	2,075297	11,6286	2,95E-31	3,35E-29	Actinomycetaceae	Arcanobacterium
		29	ASV_6	506,5535	13,28019	1,258068	10,55602	4,76E-26	2,71E-24	Lachnospiraceae	Lachnoclostridium
		30	ASV_60	30,52923	9,228449	0,945128	9,76423	1,6E-22	5,26E-21	Rikenellaceae	Alistipes
		31	ASV_63	24,51239	8,919545	0,887154	10,05411	8,81E-24	3,72E-22	Ruminococcaceae	Ruminococcaceae_UCG-005

		32	ASV_78	31,89576	9,293778	0,876395	10,60455	2,84E-26	1,75E-24	Ruminococcaceae	Candidatus_Soleaferrea
		33	ASV_81	33,87623	9,381506	0,92329	10,16096	2,96E-24	1,33E-22	Akkermansiaceae	Akkermansia
		34	ASV_99	22,6946	8,793039	0,893676	9,839181	7,63E-23	2,68E-21	Lachnospiraceae	Tyzzerella
	AUT	1	ASV_1	547,4876	8,627552	0,871212	9,902931	4,04E-23	1,61E-21	Porphyromonadaceae	Porphyromonas
		2	ASV_106	28,76153	7,788137	0,74475	10,45739	1,36E-25	9,07E-24	Ruminococcaceae	Ruminococcaceae_NK4A214_group
		3	ASV_109	20,44074	7,660682	0,771646	9,927714	3,15E-23	1,37E-21	Ruminococcaceae	Ruminococcaceae_UCG-005
		4	ASV_11	93,32562	8,767704	0,812738	10,78786	3,93E-27	3,21E-25	Ruminococcaceae	Ruminococcaceae_UCG-005
		5	ASV_12	104,4159	8,145859	0,73552	11,07497	1,66E-28	1,75E-26	Ruminococcaceae	Ruminococcaceae_UCG-005
		6	ASV_125	21,35719	-8,23623	0,589273	-13,9769	2,16E-44	3,18E-41	Sphingomonadaceae	Sphingomonas
		7	ASV_128	21,59168	7,744292	0,761409	10,17101	2,67E-24	1,41E-22	Desulfovibrionaceae	Mailhella
		8	ASV_14	58,44836	8,083865	0,784358	10,30634	6,6E-25	3,6E-23	Bacteroidaceae	Bacteroides
		9	ASV_15	62,90043	6,365362	0,614905	10,35177	4,11E-25	2,33E-23	Akkermansiaceae	Akkermansia
		10	ASV_153	41,98393	-9,42504	0,680046	-13,8594	1,12E-43	8,22E-41	Nitrospiraceae	Nitrospira
		11	ASV_158	24,04225	-9,1482	0,742153	-12,3266	6,52E-35	1,92E-32	Sphingomonadaceae	Sphingomonas
		12	ASV_21	53,36946	9,040283	0,800565	11,29238	1,43E-29	1,92E-27	Ruminococcaceae	Ruminococcaceae_UCG-005
		13	ASV_29	39,06921	8,569143	0,824431	10,394	2,64E-25	1,62E-23	Ruminococcaceae	Ruminococcaceae_UCG-002
		14	ASV_318	13,42308	-7,86015	0,665698	-11,8074	3,58E-32	8,78E-30	Steroidobacteraceae	Steroidobacter
		15	ASV_35	68,21876	7,156882	0,726233	9,854799	6,53E-23	2,47E-21	Ruminococcaceae	Ruminococcaceae_UCG-010
		16	ASV_36	39,49199	8,617626	0,758358	11,36353	6,35E-30	9,36E-28	Ruminococcaceae	Ruminococcaceae_UCG-005
		17	ASV_40	38,15451	7,135533	0,72579	9,831401	8,25E-23	3,04E-21	Acidaminococcaceae	Phascolarctobacterium
		18	ASV_44	29,47591	8,191051	0,789235	10,37847	3,11E-25	1,83E-23	Ruminococcaceae	Ruminococcaceae_UCG-009
		19	ASV_457	12,08896	-6,6171	0,57429	-11,5222	1,02E-30	1,67E-28	Dongiaceae	Dongia
		20	ASV_47	49,48487	8,933841	0,917367	9,738568	2,06E-22	7,42E-21	Rikenellaceae	Rikenellaceae_RC9_gut_group
		21	ASV_48	40,07643	8,639857	0,774499	11,15541	6,74E-29	8,27E-27	Ruminococcaceae	Ruminococcaceae_UCG-005
		22	ASV_5	145,5793	5,768417	0,49321	11,69565	1,34E-31	2,82E-29	Ruminococcaceae	Ruminococcaceae_UCG-005
		23	ASV_54	26,56016	23,59145	2,321676	10,16139	2,95E-24	1,5E-22	Clostridiaceae_1	Clostridium_sensu_stricto_1
		24	ASV_63	31,15286	8,25535	0,793511	10,40357	2,39E-25	1,53E-23	Ruminococcaceae	Ruminococcaceae_UCG-005
		25	ASV_67	25,94016	-7,66182	0,563999	-13,5848	4,93E-42	2,42E-39	Xanthobacteraceae	Bradyrhizobium
		26	ASV_76	32,07053	8,314158	0,769449	10,80533	3,25E-27	2,81E-25	Ruminococcaceae	Ruminococcaceae_UCG-005
		27	ASV_78	24,3726	7,92271	0,798702	9,919479	3,43E-23	1,44E-21	Ruminococcaceae	Candidatus_Soleaferrea
		28	ASV_98	23,83728	7,888743	0,753253	10,4729	1,15E-25	8,07E-24	Ruminococcaceae	Ruminococcaceae_UCG-005
NGRAZED vs ANIMAL	WIN	1	ASV_12	142,492	12,25491	1,195683	10,2493	1,19E-24	8,65E-22	Ruminococcaceae	Ruminococcaceae_UCG-005

		2	ASV_5	218,6043	12,8758	1,20459	10,68894	1,15E-26	1,67E-23	Ruminococcaceae	Ruminococcaceae_UCG-005
	SPR	1	ASV_11	231,3702	9,755335	0,672463	14,50687	1,1E-47	8,01E-45	Ruminococcaceae	Ruminococcaceae_UCG-005
		2	ASV_112	33,14075	9,576071	0,896188	10,68533	1,19E-26	6,7E-25	Christensenellaceae	Christensenellaceae_R-7_group
		3	ASV_114	38,05699	9,774161	0,995702	9,81635	9,57E-23	2,64E-21	Spirochaetaceae	Treponema_2
		4	ASV_115	46,84019	10,07378	0,918637	10,96601	5,57E-28	3,54E-26	Ruminococcaceae	Ruminococcaceae_UCG-005
		5	ASV_12	143,9171	11,69317	0,86169	13,57004	6,03E-42	2,94E-39	Ruminococcaceae	Ruminococcaceae_UCG-005
		6	ASV_124	43,90001	9,979691	0,978413	10,19987	1,99E-24	6,75E-23	Acidaminococcaceae	Phascolarctobacterium
		7	ASV_128	32,62044	9,549224	0,87413	10,92426	8,83E-28	5,38E-26	Desulfovibrionaceae	Mailhella
		8	ASV_129	26,06868	9,228745	0,898554	10,27066	9,55E-25	3,78E-23	Ruminococcaceae	Ruminococcaceae_UCG-002
		9	ASV_133	37,1763	9,740706	0,935809	10,40886	2,26E-25	1E-23	Bacteroidaceae	Bacteroides
		10	ASV_14	123,883	10,99554	0,890508	12,34749	5,03E-35	1,47E-32	Bacteroidaceae	Bacteroides
		11	ASV_142	31,4669	9,499525	0,973542	9,757695	1,71E-22	4,17E-21	Ruminococcaceae	Ruminococcus_1
		12	ASV_146	31,21747	9,48699	0,909621	10,4296	1,82E-25	8,3E-24	Ruminococcaceae	Ruminococcaceae_UCG-005
		13	ASV_147	25,98538	9,225826	0,906579	10,17652	2,52E-24	8,39E-23	Ruminococcaceae	Ruminococcaceae_UCG-005
		14	ASV_15	113,1256	11,34624	0,899194	12,61823	1,68E-36	6,12E-34	Akkermansiaceae	Akkermansia
		15	ASV_161	39,74572	9,837095	0,999277	9,844207	7,26E-23	2,04E-21	Bacteroidaceae	Bacteroides
		16	ASV_166	29,14009	9,38827	0,908661	10,33199	5,05E-25	2,05E-23	Lachnospiraceae	Lachnospiraceae_NK4A136_group
		17	ASV_169	42,30247	9,92618	0,972985	10,20178	1,95E-24	6,75E-23	Ruminococcaceae	Ruminococcaceae_UCG-005
		18	ASV_173	23,6837	9,091201	0,88647	10,25551	1,12E-24	4,3E-23	Ruminococcaceae	Ruminococcaceae_UCG-005
		19	ASV_182	25,25262	9,18128	0,899981	10,20163	1,95E-24	6,75E-23	Ruminococcaceae	Ruminococcaceae_UCG-010
		20	ASV_189	18,37587	8,725681	0,891181	9,791147	1,23E-22	3,15E-21	Christensenellaceae	Christensenellaceae_R-7_group
		21	ASV_206	21,70418	8,965394	0,914686	9,801606	1,11E-22	3E-21	Ruminococcaceae	Ruminococcaceae_UCG-005
		22	ASV_208	31,54097	9,502069	0,979345	9,702472	2,94E-22	6,94E-21	Bacteroidaceae	Bacteroides
		23	ASV_21	90,47127	11,02409	0,960575	11,47655	1,73E-30	2,11E-28	Ruminococcaceae	Ruminococcaceae_UCG-005
		24	ASV_212	28,51086	9,355989	0,967813	9,667141	4,16E-22	9,65E-21	Christensenellaceae	Christensenellaceae_R-7_group
		25	ASV_215	19,47781	8,804479	0,901263	9,769041	1,53E-22	3,85E-21	Family_XIII	Family_XIII_AD3011_group
		26	ASV_217	36,9444	9,7309	0,986976	9,859312	6,25E-23	1,83E-21	Lachnospiraceae	Tyzzerella_4
		27	ASV_220	23,61446	9,085131	0,930176	9,767111	1,56E-22	3,86E-21	Prevotellaceae	Prevotellaceae_UCG-003
		28	ASV_283	24,55542	9,14463	0,880016	10,39143	2,71E-25	1,17E-23	Ruminococcaceae	Ruminococcaceae_UCG-005
		29	ASV_29	44,43221	9,99845	0,9477	10,55023	5,07E-26	2,39E-24	Ruminococcaceae	Ruminococcaceae_UCG-002
		30	ASV_296	29,94465	9,427444	0,924115	10,20159	1,95E-24	6,75E-23	Lachnospiraceae	Agathobacter
		31	ASV_32	57,94222	10,38031	0,975091	10,64548	1,83E-26	9,23E-25	Prevotellaceae	Prevotellaceae_UCG-004

		32	ASV_320	37,60613	24,47967	2,154438	11,36243	6,43E-30	7,23E-28	Spirochaetaceae	Treponema_2
		33	ASV_36	63,37278	10,50975	0,894374	11,75095	6,98E-32	1,13E-29	Ruminococcaceae	Ruminococcaceae_UCG-005
		34	ASV_44	47,71882	10,10034	1,025269	9,851399	6,76E-23	1,94E-21	Ruminococcaceae	Ruminococcaceae_UCG-009
		35	ASV_48	43,27662	6,586166	0,590447	11,15455	6,8E-29	5,24E-27	Ruminococcaceae	Ruminococcaceae_UCG-005
		36	ASV_5	344,1496	12,95044	0,882633	14,67251	9,67E-49	1,41E-45	Ruminococcaceae	Ruminococcaceae_UCG-005
		37	ASV_58	54,44697	10,28971	0,871737	11,80369	3,74E-32	6,83E-30	Lachnospiraceae	Lachnoclostridium_10
		38	ASV_60	64,5337	10,0548	0,941957	10,67438	1,34E-26	7,26E-25	Rikenellaceae	Alistipes
		39	ASV_63	57,43323	10,36762	0,862507	12,02033	2,78E-33	6,77E-31	Ruminococcaceae	Ruminococcaceae_UCG-005
		40	ASV_64	69,42581	10,6407	1,071371	9,931851	3,03E-23	9,03E-22	Ruminococcaceae	Ruminococcaceae_UCG-005
		41	ASV_72	80,60156	10,85585	0,980271	11,07433	1,67E-28	1,16E-26	Spirochaetaceae	Treponema_2
		42	ASV_76	44,15921	9,990326	0,886384	11,27088	1,83E-29	1,78E-27	Ruminococcaceae	Ruminococcaceae_UCG-005
		43	ASV_78	38,98946	9,809747	0,872694	11,24076	2,57E-29	2,21E-27	Ruminococcaceae	Candidatus_Soleaferrea
		44	ASV_80	44,75599	10,00778	0,994452	10,06361	8E-24	2,54E-22	Prevotellaceae	Prevotellaceae_UCG-001
		45	ASV_87	47,34118	10,09096	0,890876	11,32701	9,64E-30	1,01E-27	Ruminococcaceae	Ruminococcaceae_UCG-009
		46	ASV_95	55,0861	10,30765	0,916319	11,24898	2,34E-29	2,14E-27	Spirochaetaceae	Treponema_2
		47	ASV_99	34,84228	9,646311	0,950846	10,14498	3,49E-24	1,13E-22	Lachnospiraceae	Tyzzerella
	SUM	1	ASV_1	1744,439	11,25888	0,864964	13,01659	9,85E-39	2,42E-36	Porphyromonadaceae	Porphyromonas
		2	ASV_102	29,41672	9,751983	0,885286	11,01562	3,21E-28	1,9E-26	Ruminococcaceae	Ruminococcaceae_UCG-010
		3	ASV_106	25,30549	9,524761	0,922059	10,32988	5,16E-25	1,86E-23	Ruminococcaceae	Ruminococcaceae_NK4A214_group
		4	ASV_109	21,952	9,328642	0,925771	10,07662	7,01E-24	2,03E-22	Ruminococcaceae	Ruminococcaceae_UCG-005
		5	ASV_11	63,13451	10,85061	0,911602	11,90279	1,14E-32	1,41E-30	Ruminococcaceae	Ruminococcaceae_UCG-005
		6	ASV_113	40,60695	10,20835	1,006076	10,1467	3,43E-24	1,08E-22	Rikenellaceae	Alistipes
		7	ASV_12	78,88384	11,1684	0,859487	12,99426	1,32E-38	2,78E-36	Ruminococcaceae	Ruminococcaceae_UCG-005
		8	ASV_124	19,75531	9,180233	0,914142	10,04246	9,92E-24	2,76E-22	Acidaminococcaceae	Phascolarctobacterium
		9	ASV_128	22,6524	9,385153	0,894534	10,49167	9,43E-26	4,5E-24	Desulfovibrionaceae	Mailhella
		10	ASV_129	18,56498	9,093751	0,881859	10,31203	6,22E-25	2,14E-23	Ruminococcaceae	Ruminococcaceae_UCG-002
		11	ASV_13	226,9293	12,68972	1,088974	11,65292	2,22E-31	2,18E-29	Campylobacteraceae	Campylobacter
		12	ASV_14	71,0437	8,596882	0,619866	13,86893	9,77E-44	4,81E-41	Bacteroidaceae	Bacteroides
		13	ASV_147	18,99198	9,126136	0,916743	9,954961	2,4E-23	6,22E-22	Ruminococcaceae	Ruminococcaceae_UCG-005
		14	ASV_148	22,90763	9,387229	0,957781	9,801014	1,11E-22	2,67E-21	Ruminococcaceae	Ruminococcaceae_UCG-005
		15	ASV_15	72,85442	11,05236	0,86977	12,70722	5,39E-37	9,95E-35	Akkermansiaceae	Akkermansia
		16	ASV_154	21,59741	9,312733	0,923721	10,08176	6,65E-24	1,97E-22	Ruminococcaceae	Ruminococcaceae_UCG-010

		17	ASV_159	21,64342	9,302367	0,895136	10,39212	2,69E-25	1,05E-23	Ruminococcaceae	Ruminococcaceae_UCG-010
		18	ASV_182	17,11392	8,980896	0,91351	9,831191	8,26E-23	2,07E-21	Ruminococcaceae	Ruminococcaceae_UCG-010
		19	ASV_189	18,70554	9,077822	0,880272	10,31252	6,19E-25	2,14E-23	Christensenellaceae	Christensenellaceae_R-7_group
		20	ASV_205	20,65007	9,234002	0,942201	9,800456	1,12E-22	2,67E-21	Christensenellaceae	Christensenellaceae_R-7_group
		21	ASV_21	34,81134	9,989989	1,022415	9,770969	1,5E-22	3,41E-21	Ruminococcaceae	Ruminococcaceae_UCG-005
		22	ASV_219	46,30862	25,0694	2,167036	11,56852	5,95E-31	5,17E-29	Spirochaetaceae	Treponema_2
		23	ASV_221	40,07339	24,96175	2,12585	11,74201	7,76E-32	8,19E-30	Lachnospiraceae	Lachnoclostridium
		24	ASV_228	16,27925	8,90576	0,888413	10,02435	1,19E-23	3,2E-22	Ruminococcaceae	Ruminococcaceae_UCG-005
		25	ASV_229	19,62128	9,159668	0,875505	10,46216	1,29E-25	5,6E-24	Ruminococcaceae	Ruminococcaceae_UCG-004
		26	ASV_26	48,23323	10,45975	1,006108	10,39625	2,58E-25	1,03E-23	Rikenellaceae	Rikenellaceae_RC9_gut_group
		27	ASV_29	52,25809	10,57865	0,867324	12,19688	3,23E-34	4,77E-32	Ruminococcaceae	Ruminococcaceae_UCG-002
		28	ASV_3	541,2238	12,09111	1,08914	11,10152	1,23E-28	7,59E-27	Spirochaetaceae	Treponema_2
		29	ASV_31	72,28912	25,26275	2,06182	12,25264	1,63E-34	2,67E-32	Dysgonomonadaceae	Proteiniphilum
		30	ASV_32	39,9208	9,712948	0,893844	10,86649	1,66E-27	9,46E-26	Prevotellaceae	Prevotellaceae_UCG-004
		31	ASV_36	39,01877	10,15506	0,862018	11,78058	4,92E-32	5,58E-30	Ruminococcaceae	Ruminococcaceae_UCG-005
		32	ASV_368	25,42043	24,49344	2,34704	10,43588	1,7E-25	6,98E-24	Deferribacteraceae	Mucispirillum
		33	ASV_4	377,0439	28,59853	2,137367	13,38026	7,89E-41	2,91E-38	Spirochaetaceae	Treponema_2
		34	ASV_40	35,96432	10,03243	0,960631	10,44358	1,57E-25	6,62E-24	Acidaminococcaceae	Phascolarctobacterium
		35	ASV_44	36,18789	10,05296	0,901698	11,14893	7,25E-29	4,65E-27	Ruminococcaceae	Ruminococcaceae_UCG-009
		36	ASV_48	33,02536	9,909688	1,014814	9,765031	1,59E-22	3,56E-21	Ruminococcaceae	Ruminococcaceae_UCG-005
		37	ASV_5	124,4975	7,14584	0,333484	21,42784	7,4E-102	1,09E-98	Ruminococcaceae	Ruminococcaceae_UCG-005
		38	ASV_52	130,9187	25,60568	1,951309	13,12231	2,45E-39	7,25E-37	Actinomycetaceae	Arcanobacterium
		39	ASV_55	115,519	24,11572	2,075297	11,62037	3,25E-31	3E-29	Actinomycetaceae	Arcanobacterium
		40	ASV_58	25,79652	9,56631	0,950177	10,06793	7,66E-24	2,18E-22	Lachnospiraceae	Lachnoclostridium_10
		41	ASV_6	506,5535	11,60544	1,135254	10,22278	1,57E-24	5,15E-23	Lachnospiraceae	Lachnoclostridium
		42	ASV_60	30,52923	9,796424	0,945128	10,36518	3,57E-25	1,35E-23	Rikenellaceae	Alistipes
		43	ASV_63	24,51239	9,48752	0,887154	10,69433	1,08E-26	5,51E-25	Ruminococcaceae	Ruminococcaceae_UCG-005
		44	ASV_71	34,4461	9,973661	1,029294	9,689805	3,33E-22	7,34E-21	Akkermansiaceae	Akkermansia
		45	ASV_76	24,69264	9,493378	0,934209	10,16194	2,93E-24	9,41E-23	Ruminococcaceae	Ruminococcaceae_UCG-005
		46	ASV_78	31,89576	9,861753	0,876395	11,25263	2,25E-29	1,58E-27	Ruminococcaceae	Candidatus_Soleaferrea
		47	ASV_81	33,87623	9,949481	0,92329	10,77612	4,46E-27	2,35E-25	Akkermansiaceae	Akkermansia
		48	ASV_98	22,39588	9,363492	0,933838	10,0269	1,16E-23	3,18E-22	Ruminococcaceae	Ruminococcaceae_UCG-005

		49	ASV_99	22,6946	9,361014	0,893676	10,47473	1,13E-25	5,21E-24	Lachnospiraceae	Tyzzerella
	AUT	1	ASV_1	547,4876	9,177426	0,917745	9,999973	1,52E-23	6,8E-22	Porphyromonadaceae	Porphyromonas
		2	ASV_106	28,76153	7,185372	0,712083	10,09064	6,08E-24	2,89E-22	Ruminococcaceae	Ruminococcaceae_NK4A214_group
		3	ASV_1091	4,486287	-6,8742	0,696651	-9,86748	5,76E-23	2,29E-21	Frankiaceae	Jatrophihabitans
		4	ASV_11	93,32562	6,295507	0,540808	11,64092	2,55E-31	3,42E-29	Ruminococcaceae	Ruminococcaceae_UCG-005
		5	ASV_125	21,35719	-8,73153	0,589417	-14,8138	1,19E-49	8,78E-47	Sphingomonadaceae	Sphingomonas
		6	ASV_14	58,44836	4,678211	0,42815	10,92656	8,6E-28	9,05E-26	Bacteroidaceae	Bacteroides
		7	ASV_153	41,98393	-9,39353	0,680617	-13,8015	2,5E-43	1,23E-40	Nitrospiraceae	Nitrospira
		8	ASV_158	24,04225	-7,98724	0,745112	-10,7195	8,24E-27	6,39E-25	Sphingomonadaceae	Sphingomonas
		9	ASV_21	53,36946	4,842171	0,461517	10,49186	9,42E-26	5,55E-24	Ruminococcaceae	Ruminococcaceae_UCG-005
		10	ASV_288	9,378521	-7,55851	0,74753	-10,1113	4,92E-24	2,42E-22	Chthoniobacteraceae	Candidatus_Udaeobacter
		11	ASV_307	11,83643	-8,32426	0,800774	-10,3953	2,61E-25	1,45E-23	Dongiaceae	Dongia
		12	ASV_318	13,42308	-7,80388	0,667545	-11,6904	1,43E-31	2,1E-29	Steroidobacteraceae	Steroidobacter
		13	ASV_36	39,49199	7,99219	0,740347	10,7952	3,63E-27	3,14E-25	Ruminococcaceae	Ruminococcaceae_UCG-005
		14	ASV_374	18,74163	-9,10917	0,912384	-9,98392	1,79E-23	7,77E-22	Nitrosomonadaceae	IS-44
		15	ASV_40	38,15451	5,644906	0,518387	10,88936	1,3E-27	1,19E-25	Acidaminococcaceae	Phascolarctobacterium
		16	ASV_457	12,08896	-7,64101	0,572341	-13,3504	1,18E-40	3,47E-38	Dongiaceae	Dongia
		17	ASV_474	4,185265	-6,45969	0,59153	-10,9203	9,22E-28	9,05E-26	Streptomycetaceae	Streptomyces
		18	ASV_48	40,07643	8,010995	0,756956	10,58318	3,57E-26	2,39E-24	Ruminococcaceae	Ruminococcaceae_UCG-005
		19	ASV_537	6,879708	-7,3151	0,609357	-12,0046	3,36E-33	6,19E-31	Reyranellaceae	Reyranella
		20	ASV_54	26,56016	23,4082	2,321676	10,08246	6,61E-24	3,04E-22	Clostridiaceae_1	Clostridium_sensu_stricto_1
		21	ASV_597	4,178363	-6,88632	0,704064	-9,78082	1,36E-22	5,28E-21	Pseudonocardiaceae	Pseudonocardia
		22	ASV_640	11,88781	-8,05705	0,824923	-9,76704	1,56E-22	5,89E-21	Nitrosomonadaceae	Ellin6067
		23	ASV_67	25,94016	-8,66288	0,563143	-15,3831	2,13E-53	3,13E-50	Xanthobacteraceae	Bradyrhizobium
		24	ASV_686	8,195678	-7,70156	0,744675	-10,3422	4,54E-25	2,39E-23	Nitrosomonadaceae	MND1
		25	ASV_76	32,07053	7,338859	0,738416	9,938651	2,83E-23	1,19E-21	Ruminococcaceae	Ruminococcaceae_UCG-005
		26	ASV_98	23,83728	7,264536	0,735086	9,882563	4,95E-23	2,03E-21	Ruminococcaceae	Ruminococcaceae_UCG-005
