# Supplementary material for: Seasonal dynamics in sheep fecal microbiome and soil bacterial communities under grazing management
Source: PLoS One. 2026 Jun 29;21(6):e0352436. doi: 10.1371/journal.pone.0352436 (PMC13313378; doi:10.1371/journal.pone.0352436)
Supplement: Attachment — Submitted filename: Response_to_reviewers_auresp_2.docx [file pone.0352436.s008.docx]

We would like to thank the Editor for considering our manuscript for publication and the Reviewer for their careful evaluation of our work, as well as for the constructive comments and suggestions. We have revised the manuscript accordingly and believe that these changes have improved its clarity and overall quality. Below, we provide a detailed, point-by-point response to each comment.

Reviewer #2: I would like to thank the authors for carefully and thoroughly addressing all the comments, and I congratulate them on the high quality of their revision and the great effort. I have only a few minor remarks that the authors should address.

Line 59: Please replace the term “longitudinal studies” with “over‑time studies” to maintian consistency in terminology and methodology. Please also review the corresponding citation if appropriate.

A: Changed (L59)

Please explicitly refer to the study design as “repeated cross‑sectional” rather than merely “cross‑sectional” wherever it appears in the manuscript, as the “repeated cross‑sectional” designation clearly reflects an additional parameter (seasons, in this case).

A: The term has been changed in the whole manuscript: L40, L385

Line 113: I suggested reformulating “with a Ap-Bt-2Btk horizons succession” to “with an Ap–Bt–2Btk horizon sequence” because the latter phrasing is more consistent with standard soil-profile terminology, where “horizon sequence” is conventionally used to describe the vertical arrangement of diagnostic horizons. Please update the expression to improve clarity and terminological accuracy.

A: The term “Ap-Bt-2Btk horizons succession“ has been updated (L114).

In my previous comment, I requested additional methodological clarifications to improve transparency and reproducibility, including clarification of whether biological or technical replicates were included for soil and fecal samples, and a brief justification for selecting lactating ewes as the study population. The authors have addressed these points in their response to reviewers, but the corresponding explanations have not yet been incorporated into the manuscript text. Please add clear, concise statements in the Methods (or relevant section) explicitly stating the use of biological replicates for soil and fecal samples, and the rationale for choosing lactating ewes as the study population.

A: Clarifications regarding the use of biological replicates and the rationale for selecting lactating ewes have been added to the Methods section (L155–158).
