# Supplementary material for: Seasonal dynamics in sheep fecal microbiome and soil bacterial communities under grazing management
Source: PLoS One. 2026 Jun 29;21(6):e0352436. doi: 10.1371/journal.pone.0352436 (PMC13313378; doi:10.1371/journal.pone.0352436)
Supplement: Attachment — Submitted filename: Response_to_reviewers.docx [file pone.0352436.s007.docx]

We would like to thank the Editor for considering our manuscript for publication and the Reviewers for their careful evaluation of our work, as well as for their constructive comments and suggestions. We have revised the manuscript accordingly and believe that these changes have improved its clarity and overall quality. Below, we provide a detailed, point-by-point response to each comment.

Reviewer #1: The manuscript is very interesting and well written, all the research points are well investigated and discussed. The topic is new and relevant and I only have some small format issues to present.

The main issue is the quality of the figures that seems low, can you please check? In the taxa abundance bar plots I suggest to keep in the legend only the taxa that are clearly visible in the figures to reduce the use of similar colors and I personally would avoid manteining taxa identified by a code (eg. Family XI in Fig.3 can be eliminated, also considering that is not discussed, as well as Clostridiales_vadinBB60_group can be called simply Clostridiales).

A: Thank you for your comments. Regarding the quality of the figures, we would like to clarify that all figures meet the journal’s requirement of at least 300 dpi resolution. Nevertheless, we carefully rechecked the files to ensure optimal quality in the submitted version.

Concerning the taxa abundance bar plots, we already applied a filtering criterion to improve readability: only phyla, families, and genera with relative abundance higher than 3% were included in the plots. We chose this threshold as a compromise between clarity and information content. Increasing this cutoff further would substantially reduce the amount of information conveyed by the figures.

Line 83 pg. 4: Please note that it should be bacterial and fungal taxa and not bacteria and fungi taxa.

A: Changed (L88).

Line 154 pg. 7: Please check the total number of soil samples.

A: Checked and confirmed.

Line 180 pg. 8: Please specify which version of Silva has been used.

A: SILVA version was added (L200).

Lines 233-235 pg. 11: Please rephrase for better clarity and fluency.

A: Rephrased (L274)

Lines 236-239: It seems to me that these changes are mainly in NGRAZED, correct? If so, please specify.

A: Authors checked and confirm the sentence.

Lines 247-249: Please rephrase for better clarity and fluency.

A: Rephrased (L289-290)

Line 263 pg. 12: Which is the difference between Ruminococcaceae_UCG.005 and Ruminococcaceae_UCG.010? I suggest to you to consider both taxa as Ruminococcaceae since there are not reported in the text information on their taxonomic difference.

A: Modified L307-306.

Lines 287-289 pg. 13: Can you please explain why PCA values are so small? It is because of the high variability in animal samples that is cited on lines 296-300?

A: Thank you for this comment. The relatively low percentage of variance explained by the first axes of the PCoA reflects the high compositional complexity and variability typical of microbiome datasets. In such datasets, community differences are often distributed across many dimensions rather than concentrated in the first few axes. The combined dataset including both sheep fecal samples and soil samples surely amplify the heterogeneous microbial communities. This increases overall beta-diversity and spreads the variance across multiple coordinates, resulting in lower percentages explained by the first axes. We have clarified this point in the revised manuscript (lines 351) to better explain that the low variance values are expected in highly diverse microbial datasets and do not affect the interpretation of the observed clustering patterns.

Reviewer #2: This manuscript investigates the relationship between sheep gut microbiota and soil microbial communities under grazing management across seasons, using fecal sampling from lactating ewes and soil sampling from grazed and ungrazed areas. The study applies 16S rRNA sequencing, diversity analyses, and differential abundance testing to explore how grazing influences microbial composition. The topic is timely and the dataset valuable; however, several aspects of the experimental design, statistical analysis, and interpretation of results warrant clarification. In the following comments, I provide specific suggestions on methodology, data presentation, and discussion points to improve transparency, rigor, and interpretability of the study.

Lines 53 – 56: I recommend explicitly referring to gut microbiota (or fecal microbiota, as appropriate) to improve conceptual clarity, be more specific, and avoid ambiguity.

A: Modified (L54)

Line 59: Please, consider rephrasing. I suggest “Collection of post mortem gut contents and mucosal scrapings, which precludes longitudinal studies (ref); endoscopic or biopsy-based sampling, which is technically challenging in large animals and often requires anesthesia or surgical intervention”

A: Modified (L61-64).

Line 66: As fecal sampling does not fully mirror the rumen microbial community and primarily reflects the distal gut environment, presenting it as a complete proxy for the entire gastrointestinal microbiota may be overstated. I recommend softening the statement to acknowledge this limitation. For example, fecal microbiota analyses can provide valuable insights into aspects of animal health.

A: Pointed out (L71).

Lines 89-90: The statement “extensive sheep grazing may represent a valuable approach that addresses all these concerns” may be somewhat overstated. While sheep grazing can contribute to sustainability, animal welfare, and mitigation of rural abandonment, it may be more accurate to frame these outcomes as potential benefits rather than definitive solutions. Given the scope of the present study, causal relationships between grazing and these broader socio-ecological outcomes cannot be established. I suggest moderating the language to reflect contribution or association rather than direct resolution.

A: We agree that the original wording could be interpreted as overstating the role of extensive sheep grazing. The sentence has been revised to moderate the claim and to emphasize its potential contribution rather than a definitive solution.

Line 91: The study design appears to be repeated cross-sectional rather than a true longitudinal setup, as different individuals were sampled across seasons rather than following the same animals longitudinally. I recommend stating this explicitly in the abstract, Methods section, Discussion, and Conclusion to avoid ambiguity. Additionally, the implications of this design should be acknowledged, as seasonal differences cannot be interpreted as within-individual temporal changes but rather as population-level trends. Clarifying this distinction would strengthen the methodological transparency of the study.

A: Modified in the whole manuscript and clarified in all sections.

Lines 110-111: I suggest reformulating “with a Ap-Bt-2Btk horizons succession” to “with an Ap-Bt-2Btk horizon sequence”.

A: Changed at L122.

Lines 112-113: Could the authors please clarify what is meant by “with typically 120 -150 ewes in lactation contemporarily”?

A: We have clarified this point in the revised manuscript (L124).

Lines 149-156: I recommend providing additional methodological details to improve clarity and reproducibility. (i) Specifically, for soil sampling, please clarify how random sampling points were selected, indicate the sampling depth, and specify whether samples were collected from bulk soil or rhizospheric zones. (ii) Additionally, the rationale for uneven soil sample numbers across seasons should be explained. (iii) For both soil and fecal samples, please clarify whether biological or technical replicates were included. (iv) It would also strengthen the manuscript to briefly justify the choice of lactating ewes as the study population. (v) In line 155, “transfer” should be corrected to “transferred”.

A: Authors answered below points by points and specified in the manuscript:

i) We have clarified that soil samples were collected from multiple locations distributed across the GRAZED and NGRAZED perimeters to capture spatial variability. Although a formal coordinate-based randomization scheme was not applied, sampling points were selected across the entire area while avoiding immediate proximity between collection points. Sampling depth was 10 cm from bulk soil. All these info were integrated in the manuscript.

(ii) The number of soil samples varied across seasons because the sampling effort was progressively expanded as additional funding became available during the study. A sentence was added at L169-170.

(iii) all samples represent biological replicates, with no technical replicates included.

(iv) they represent a metabolically homogeneous and management-relevant group within the flock.

(v) Corrected.

Line 171: The Methods state that forward reads were truncated at 300 bp; since this corresponds to the full sequenced read length, it might be clearer to state that the forward reads were retained at full length, and truncation was applied only to reverse reads at 280 bp.

A: We agree that the original wording could be unclear. The sentence has been revised to clarify that forward reads were retained at their full length, while truncation was applied only to reverse reads L191)

Line 181: Please provide the version of the vegan package as well as the versions of all other R packages used in the analyses.

A: All R packages versions have been added.

Lines 183-185: The manuscript indicates that GRAZED and NGRAZED soils were compared using a paired t-test. However, the experimental design does not clearly describe a true pairing structure between samples. Since a paired t-test assumes a natural one-to-one correspondence between observations, clarification of the pairing rationale is needed. If samples were independent, an unpaired t-test may have been more appropriate.

A: Authors thank the reviewer for this important comment and they have corrected this sentence in the revised manuscript by applying a two-tailed independent samples t-test (L204).

Lines 188-191: The Kruskal–Wallis test was used to assess associations between categorical groups (ANIMAL, GRAZED, NGRAZED) and alpha diversity estimates among the four seasons. However, it is unclear whether season was incorporated as an independent factor in the analysis. I kindly suggest clarifying the statistical modeling approach to handle season.

A: Authors agree that this was not clearly described in the original manuscript. The M&M section has now been revised to explicitly clarify that analyses were conducted independently for each season (L208-211).

Lines194-197: It is unclear whether the assumptions of PERMANOVA, particularly the homogeneity of group dispersions, were assessed. Differences in dispersion can bias significance. I suggest clarifying whether dispersion was checked.

A: We thank the reviewer for this important comment. We agree that assessing the homogeneity of multivariate dispersions is essential for a correct interpretation of PERMANOVA results.

We have now performed a PERMDISP analysis (using the betadisper function in the vegan R package) for each season. Results showed that dispersion was not significantly different among groups in Winter (p = 0.193) and Autumn (p = 0.459), whereas significant differences were detected in Spring (p = 0.008) and Summer (p = 0.001). The Materials and Methods and Results sections have been updated accordingly, as well as Discussion (e.g. L221, L575-579).

Lines 198-203: The manuscript reports differential abundance analysis using DESeq2 with significance defined by both FDR ≤ 0.05 and fold-change thresholds (>1.5 or < -1.5), and additionally applies Bonferroni-adjusted p-value cutoffs that differ between soil (≤ 0.01) and ANIMAL samples (≤ 1×10⁻²⁰). The rationale for using both FDR and Bonferroni corrections, as well as the extremely stringent threshold for the ANIMAL group, is unclear. I suggest clarifying the justification for these choices and ensuring consistency in multiple-testing correction, so that the criteria for identifying significant taxa are transparent and biologically interpretable.

A: Differential abundance was primarily assessed with false discovery rate (FDR) correction (Benjamini–Hochberg), and taxa were considered significant when FDR ≤ 0.05 together with a fold-change threshold (|log2FC| corresponding to >1.5 or < −1.5 to ensure biological relevance. The additional Bonferroni-adjusted p-value thresholds were applied only as a more conservative filter for reporting the most robust differences and for visualization purposes. Because the number of significant taxa detected in ANIMAL samples was substantially higher than in soil samples, a more stringent cutoff was applied to highlight the most strongly differentiated taxa and improve figure readability. To avoid confusion, we have now clarified the rationale for these thresholds in the Methods section (L225-232).

Line 208: In the Results section, the authors report summary statistics for soil physico-chemical parameters; however, the individual replicate values are not provided. Given the small sample size (n = 5 per area), access to raw data (e.g., in supplementary material) would improve transparency.

A: Data was included in Supplementary Table 1.

Table 1: Please clarify the meaning of the parenthetical values in the table and explain their absence for the clay, silt, and sand measurements.

Values in parentheses indicate standard deviations. Soil texture was determined on a pooled sample obtained by bulking all individual samples, and the analysis was performed in laboratory duplicate. Therefore, a single value without standard deviation is reported for texture. This information has now been added to the manuscript (L245).

Line 213: Please write out "Soil Organic Matter (SOM)" in full the first time it is mentioned, and subsequently use the abbreviation "SOM" if needed.

A: Changed.

Line 219: Numerical data summarizing the distribution of ASVs by group at the phylum, family, and genus levels are not provided. Providing such counts or relative abundances in a supplementary table would greatly improve transparency and allow readers to evaluate the contribution of dominant and rare taxa to observed patterns. I recommend that the authors provide a detailed table or supplementary dataset summarizing ASV counts or relative abundances by group and season.

A: We have provided a supplementary table containing, for each sample, the ASV-level relative abundances together with the corresponding alpha diversity indices (observed richness S.obs and Shannon index shannon), as well as sample metadata including group (GRAZED, NGRAZED, ANIMAL) and season (AUT, WIN, SPR, SUM). This table allows readers to examine the full set of alpha diversity estimates and evaluate the contribution of each ASV to community composition across groups and seasons.

Line 272: The manuscript reports alpha diversity results using Shannon and Chao1 indices by some key values, but the individual numerical values for each sample are not provided. I recommend that the authors provide the full set of alpha diversity estimates per sample, along with the corresponding group and season information.

A: We have provided a supplementary table containing all the requested information.

Table 2: The table presents PERMANOVA results in a raw-output style, which may be difficult for some readers to interpret. I suggest revising the table title to clearly indicate what is being tested and how. Additionally, consider adding brief footnotes explaining the columns (e.g., R², Residual), highlighting significant results, and formatting p-values and effect sizes for readability.

A: Modifications have been done to better interpret results.

Lines 306-313: The results report differential abundance using only the most significant non-overlapping ASVs shown at the Family level. I recommend that the authors provide the full set of ASV-level results, including all significant taxa across seasons, and clarify the choice of thresholds.

A: Details on thresholds have been added, as requested above. S4 Table reports now the list of significant taxa, as the reviewer suggests.

Lines 365-367: In the Discussion, the authors state that similar physico-chemical properties between grazed and non-grazed soils support the reliability of subsequent microbial analyses, while this may be overstated, because this claim is true for bulk soil characteristics, the depth and sampling compartment (bulk vs. rhizosphere) were not specified. Given that sheep interact mainly with aboveground biomass and roots, soil microbial communities in the root zone may differ even when bulk properties are similar. I recommend clarifying the sampling depth and soil compartment, and discussing how plant-soil interactions and grazing could influence microbial composition, to contextualize links between sheep gut microbiota and soil communities more accurately.

A: We thank the reviewer for this comment. Soil samples were collected from the top 10 cm of bulk soil (the information is now clearly described in main text). We also revised the Discussion to moderate the original statement and avoid overinterpretation. Specifically, we now clarify that the similarity observed between GRAZED and NGRAZED soils refers only to the measured bulk soil physico-chemical characteristics, and therefore does not exclude the possibility that other unmeasured compartments, such as the rhizospher, may differ between plots. In addition, we now acknowledge that grazing-related plant–soil interactions, including effects on vegetation and root-associated microbial communities, may also have contributed to the observed microbial patterns, but these aspects were not directly assessed in the present study. Finally, this limitation has also been clarified in the Conclusions section.

Line 419: In the statement that “grazing appears to inhibit dominant groups such as Cyanobacteria” , the term “inhibit” is strong and involve a mechanistic investigation. The study reports relative abundances, and causation or direct inhibition cannot be inferred from observational data alone. I recommend rephrasing with more cautious descriptive terms, for example, indicating that grazing can negatively influence the abundance of … , so just a potential effect not a confirmed causation of a mechanistic phenomenon.

A: Modified (L521)

Lines 543-544: The statement that differential abundance of ASVs between grazed and ungrazed soils “supports the hypothesis of fecal microbiota being transferred to the soil” may overstate the findings. The data show associations between grazing and soil microbial composition, but direct evidence of microbial transfer from feces to soil was not measured as it requires tracking approach. I recommend rephrasing to more cautious statement, such as indicating that observed differences are consistent with a potential influence of fecal microbiota or that grazing may alter soil microbial communities, potentially including contributions from fecal input. please see: https://doi.org/10.1371/journal.pone.0316616

A: Modified L612

Line 560: The study’s limitations should be acknowledged, please state them in the conclusion. For example, repeated cross-sectional design instead of true longitudinal study, which limits the causality inference; only lactating ewes were sampled, and soil depth and compartment (bulk vs. rhizosphere) were not specified, which may affect links between gut and soil microbiota. The observational design precludes causal inferences between grazing, animal microbiota, and soil microbial changes. Additionally, differences in vegetation structure and seasonal variability may independently influence soil microbial communities.

A: We have now expanded the Conclusions section to explicitly acknowledge the main study limitations cited by the reviewers (L637-642)

Finally, I recommend that the authors make the raw sequencing data and ASV tables publicly available in a recognized repository to ensure transparency and reproducibility.

A: The raw ASVs data have been uploaded on Dryad repository. Data will be available after paper acceptance at <https://datadryad.org/dataset/doi:10.5061/dryad.hdr7sqvzh> and immediately available here for review: https://datadryad.org/share/LINK_NOT_FOR_PUBLICATION/hfEsdpVds3Jw1K1jblg7pVmT0fB9SvIGMl6I-KOdqTU
