# Supplementary material for: Seasonal dynamics in sheep fecal microbiome and soil bacterial communities under grazing management
Source: PLoS One. 2026 Jun 29;21(6):e0352436. doi: 10.1371/journal.pone.0352436 (PMC13313378; doi:10.1371/journal.pone.0352436)
Supplement: Peer review history

# Peer review: Decision Letter 0

**DOI:** 10.1371/journal.pone.0352436.r001

**Authors:** Raffaella Balestrini

13 Mar 2026

PONE-D-25-59668

Seasonal dynamics in sheep fecal microbiome and soil bacterial communities under grazing management

PLOS One

Dear Dr. Fabbri,

Thank you for submitting your manuscript to PLOS ONE. After careful consideration, we feel that it has merit but does not fully meet PLOS ONE’s publication criteria as it currently stands. Therefore, we invite you to submit a revised version of the manuscript that addresses the points raised during the review process.

Please submit your revised manuscript by Apr 27 2026 11:59PM. If you will need more time than this to complete your revisions, please reply to this message or contact the journal office at plosone@plos.org. When you're ready to submit your revision, log on to https://www.editorialmanager.com/pone/ and select the 'Submissions Needing Revision' folder to locate your manuscript file.

Please include the following items when submitting your revised manuscript:

- A letter that responds to each point raised by the academic editor and reviewer(s). You should upload this letter as a separate file labeled 'Response to Reviewers'.
- A marked-up copy of your manuscript that highlights changes made to the original version. You should upload this as a separate file labeled 'Revised Manuscript with Track Changes'.
- An unmarked version of your revised paper without tracked changes. You should upload this as a separate file labeled 'Manuscript'.

-->If you would like to make changes to your financial disclosure, please include your updated statement in your cover letter. Guidelines for resubmitting your figure files are available below the reviewer comments at the end of this letter.

If applicable, we recommend that you deposit your laboratory protocols in protocols.io to enhance the reproducibility of your results. Protocols.io assigns your protocol its own identifier (DOI) so that it can be cited independently in the future. For instructions see: https://journals.plos.org/plosone/s/submission-guidelines#loc-laboratory-protocols. Additionally, PLOS ONE offers an option for publishing peer-reviewed Lab Protocol articles, which describe protocols hosted on protocols.io. Read more information on sharing protocols at https://plos.org/protocols?utm_medium=editorial-email&utm_source=authorletters&utm_campaign=protocols.

We look forward to receiving your revised manuscript.

Kind regards,

Raffaella Balestrini

Academic Editor

PLOS One

Journal Requirements:

When submitting your revision, we need you to address these additional requirements.

1. Please ensure that your manuscript meets PLOS ONE's style requirements, including those for file naming. The PLOS ONE style templates can be found at

https://journals.plos.org/plosone/s/file?id=wjVg/PLOSOne_formatting_sample_main_body.pdf and https://journals.plos.org/plosone/s/file?id=ba62/PLOSOne_formatting_sample_title_authors_affiliations.pdf

2. Please note that PLOS One has specific guidelines on code sharing for submissions in which author-generated code underpins the findings in the manuscript. In these cases, we expect all author-generated code to be made available without restrictions upon publication of the work. Please review our guidelines at https://journals.plos.org/plosone/s/materials-and-software-sharing#loc-sharing-code and ensure that your code is shared in a way that follows best practice and facilitates reproducibility and reuse.

3. In your Methods section, please provide additional information regarding the permits you obtained for the work. Please ensure you have included the full name of the authority that approved the field site access and, if no permits were required, a brief statement explaining why.

4. Your ethics statement should only appear in the Methods section of your manuscript. If your ethics statement is written in any section besides the Methods, please delete it from any other section.

5. We note that you have indicated that there are restrictions to data sharing for this study. For studies involving human research participant data or other sensitive data, we encourage authors to share de-identified or anonymized data. However, when data cannot be publicly shared for ethical reasons, we allow authors to make their data sets available upon request. For information on unacceptable data access restrictions, please see http://journals.plos.org/plosone/s/data-availability#loc-unacceptable-data-access-restrictions.

Before we proceed with your manuscript, please address the following prompts:

a) If there are ethical or legal restrictions on sharing a de-identified data set, please explain them in detail (e.g., data contain potentially identifying or sensitive patient information, data are owned by a third-party organization, etc.) and who has imposed them (e.g., a Research Ethics Committee or Institutional Review Board, etc.). Please also provide contact information for a data access committee, ethics committee, or other institutional body to which data requests may be sent.

b) If there are no restrictions, please upload the minimal anonymized data set necessary to replicate your study findings to a stable, public repository and provide us with the relevant URLs, DOIs, or accession numbers. Please see http://www.bmj.com/content/340/bmj.c181.long for guidelines on how to de-identify and prepare clinical data for publication. For a list of recommended repositories, please see https://journals.plos.org/plosone/s/recommended-repositories. You also have the option of uploading the data as Supporting Information files, but we would recommend depositing data directly to a data repository if possible.

Please update your Data Availability statement in the submission form accordingly.

6. In the online submission form, you indicated that the data of this study are freely available from the corresponding author upon request. The data are not publicly available due to the involvement of private partners (farmers).

All PLOS journals now require all data underlying the findings described in their manuscript to be freely available to other researchers, either 1. In a public repository, 2. Within the manuscript itself, or 3. Uploaded as supplementary information.

This policy applies to all data except where public deposition would breach compliance with the protocol approved by your research ethics board. If your data cannot be made publicly available for ethical or legal reasons (e.g., public availability would compromise patient privacy), please explain your reasons on resubmission and your exemption request will be escalated for approval.

7. We note that the grant information you provided in the ‘Funding Information’ and ‘Financial Disclosure’ sections do not match.

When you resubmit, please ensure that you provide the correct grant numbers for the awards you received for your study in the ‘Funding Information’ section.

8. Thank you for stating the following financial disclosure:

European Union Next-GenerationEU (PIANO NAZIONALE DI RIPRESA E RESILIENZA (PNRR) – MISSIONE 4 COMPONENTE 2, INVESTIMENTO 1.4 – D.D. 1032 17/06/2022, CN00000022)

Please state what role the funders took in the study. If the funders had no role, please state: "The funders had no role in study design, data collection and analysis, decision to publish, or preparation of the manuscript."

If this statement is not correct you must amend it as needed.

Please include this amended Role of Funder statement in your cover letter; we will change the online submission form on your behalf.

9. We note that Figure 1 in your submission contain [map/satellite] images which may be copyrighted. All PLOS content is published under the Creative Commons Attribution License (CC BY 4.0), which means that the manuscript, images, and Supporting Information files will be freely available online, and any third party is permitted to access, download, copy, distribute, and use these materials in any way, even commercially, with proper attribution. For these reasons, we cannot publish previously copyrighted maps or satellite images created using proprietary data, such as Google software (Google Maps, Street View, and Earth). For more information, see our copyright guidelines: http://journals.plos.org/plosone/s/licenses-and-copyright.

We require you to either (1) present written permission from the copyright holder to publish these figures specifically under the CC BY 4.0 license, or (2) remove the figures from your submission:

a. You may seek permission from the original copyright holder of Figure 1 to publish the content specifically under the CC BY 4.0 license.

We recommend that you contact the original copyright holder with the Content Permission Form (http://journals.plos.org/plosone/s/file?id=7c09/content-permission-form.pdf) and the following text:

“I request permission for the open-access journal PLOS ONE to publish XXX under the Creative Commons Attribution License (CCAL) CC BY 4.0 (http://creativecommons.org/licenses/by/4.0/). Please be aware that this license allows unrestricted use and distribution, even commercially, by third parties. Please reply and provide explicit written permission to publish XXX under a CC BY license and complete the attached form.”

Please upload the completed Content Permission Form or other proof of granted permissions as an "Other" file with your submission.

In the figure caption of the copyrighted figure, please include the following text: “Reprinted from [ref] under a CC BY license, with permission from [name of publisher], original copyright [original copyright year].”

b. If you are unable to obtain permission from the original copyright holder to publish these figures under the CC BY 4.0 license or if the copyright holder’s requirements are incompatible with the CC BY 4.0 license, please either i) remove the figure or ii) supply a replacement figure that complies with the CC BY 4.0 license. Please check copyright information on all replacement figures and update the figure caption with source information. If applicable, please specify in the figure caption text when a figure is similar but not identical to the original image and is therefore for illustrative purposes only.

The following resources for replacing copyrighted map figures may be helpful:

USGS National Map Viewer (public domain): http://viewer.nationalmap.gov/viewer/

The Gateway to Astronaut Photography of Earth (public domain): http://eol.jsc.nasa.gov/sseop/clickmap/

Maps at the CIA (public domain): https://www.cia.gov/library/publications/the-world-factbook/index.html and https://www.cia.gov/library/publications/cia-maps-publications/index.html

NASA Earth Observatory (public domain): http://earthobservatory.nasa.gov/

Landsat: http://landsat.visibleearth.nasa.gov/

USGS EROS (Earth Resources Observatory and Science (EROS) Center) (public domain): http://eros.usgs.gov/#

Natural Earth (public domain): http://www.naturalearthdata.com/

10. If the reviewer comments include a recommendation to cite specific previously published works, please review and evaluate these publications to determine whether they are relevant and should be cited. There is no requirement to cite these works unless the editor has indicated otherwise.

11. Please review your reference list to ensure that it is complete and correct. If you have cited papers that have been retracted, please include the rationale for doing so in the manuscript text, or remove these references and replace them with relevant current references. Any changes to the reference list should be mentioned in the rebuttal letter that accompanies your revised manuscript. If you need to cite a retracted article, indicate the article’s retracted status in the References list and also include a citation and full reference for the retraction notice.

[Note: HTML markup is below. Please do not edit.]

Reviewers' comments:

Reviewer's Responses to Questions

**Comments to the Author**

1. Is the manuscript technically sound, and do the data support the conclusions?

The manuscript must describe a technically sound piece of scientific research with data that supports the conclusions. Experiments must have been conducted rigorously, with appropriate controls, replication, and sample sizes. The conclusions must be drawn appropriately based on the data presented.

Reviewer #1: Yes

Reviewer #2: Partly

**********

2. Has the statistical analysis been performed appropriately and rigorously?

Reviewer #1: Yes

Reviewer #2: Yes

**********

3. Have the authors made all data underlying the findings in their manuscript fully available?

The PLOS Data policy requires authors to make all data underlying the findings described in their manuscript fully available without restriction, with rare exception (please refer to the Data Availability Statement in the manuscript PDF file). The data should be provided as part of the manuscript or its supporting information, or deposited to a public repository. For example, in addition to summary statistics, the data points behind means, medians and variance measures should be available. If there are restrictions on publicly sharing data—e.g. participant privacy or use of data from a third party—those must be specified.

Reviewer #1: Yes

Reviewer #2: No

**********

4. Is the manuscript presented in an intelligible fashion and written in standard English?

PLOS ONE does not copyedit accepted manuscripts, so the language in submitted articles must be clear, correct, and unambiguous. Any typographical or grammatical errors should be corrected at revision, so please note any specific errors here.

Reviewer #1: Yes

Reviewer #2: Yes

**********

5. Review Comments to the Author

Please use the space provided to explain your answers to the questions above. You may also include additional comments for the author, including concerns about dual publication, research ethics, or publication ethics. (Please upload your review as an attachment if it exceeds 20,000 characters)

Reviewer #1: The manuscript is very interesting and well written, all the research points are well investigated and discussed. The topic is new and relevant and I only have some small format issues to present.

The main issue is the quality of the figures that seems low, can you please check? In the taxa abundance bar plots I suggest to keep in the legend only the taxa that are clearly visible in the figures to reduce the use of similar colors and I personally would avoid manteining taxa identified by a code (eg. Family XI in Fig.3 can be eliminated, also considering that is not discussed, as well as Clostridiales_vadinBB60_group can be called simply Clostridiales).

Line 83 pg. 4: Please note that it should be bacterial and fungal taxa and not bacteria and fungi taxa.

Line 154 pg. 7: Please check the total number of soil samples.

Line 180 pg. 8: Please specify which version of Silva has been used.

Lines 233-235 pg. 11: Please rephrase for better clarity and fluency.

Lines 236-239: It seems to me that these changes are mainly in NGRAZED, correct? If so, please specify.

Lines 247-249: Please rephrase for better clarity and fluency.

Line 263 pg. 12: Which is the difference between Ruminococcaceae_UCG.005 and Ruminococcaceae_UCG.010? I suggest to you to consider both taxa as Ruminococcaceae since there are not reported in the text information on their taxonomic difference.

Lines 287-289 pg. 13: Can you please explain why PCA values are so small? It is because of the high variability in animal samples that is cited on lines 296-300?

Reviewer #2: This manuscript investigates the relationship between sheep gut microbiota and soil microbial communities under grazing management across seasons, using fecal sampling from lactating ewes and soil sampling from grazed and ungrazed areas. The study applies 16S rRNA sequencing, diversity analyses, and differential abundance testing to explore how grazing influences microbial composition. The topic is timely and the dataset valuable; however, several aspects of the experimental design, statistical analysis, and interpretation of results warrant clarification. In the following comments, I provide specific suggestions on methodology, data presentation, and discussion points to improve transparency, rigor, and interpretability of the study.

Lines 53 – 56: I recommend explicitly referring to gut microbiota (or fecal microbiota, as appropriate) to improve conceptual clarity, be more specific, and avoid ambiguity.

Line 59: Please, consider rephrasing. I suggest “Collection of post mortem gut contents and mucosal scrapings, which precludes longitudinal studies (ref); endoscopic or biopsy-based sampling, which is technically challenging in large animals and often requires anesthesia or surgical intervention”

Line 66: As fecal sampling does not fully mirror the rumen microbial community and primarily reflects the distal gut environment, presenting it as a complete proxy for the entire gastrointestinal microbiota may be overstated. I recommend softening the statement to acknowledge this limitation. For example, fecal microbiota analyses can provide valuable insights into aspects of animal health.

Lines 89-90: The statement “extensive sheep grazing may represent a valuable approach that addresses all these concerns” may be somewhat overstated. While sheep grazing can contribute to sustainability, animal welfare, and mitigation of rural abandonment, it may be more accurate to frame these outcomes as potential benefits rather than definitive solutions. Given the scope of the present study, causal relationships between grazing and these broader socio-ecological outcomes cannot be established. I suggest moderating the language to reflect contribution or association rather than direct resolution.

Line 91: The study design appears to be repeated cross-sectional rather than a true longitudinal setup, as different individuals were sampled across seasons rather than following the same animals longitudinally. I recommend stating this explicitly in the abstract, Methods section, Discussion, and Conclusion to avoid ambiguity. Additionally, the implications of this design should be acknowledged, as seasonal differences cannot be interpreted as within-individual temporal changes but rather as population-level trends. Clarifying this distinction would strengthen the methodological transparency of the study.

Lines 110-111: I suggest reformulating “with a Ap-Bt-2Btk horizons succession” to “with an Ap-Bt-2Btk horizon sequence”.

Lines 112-113: Could the authors please clarify what is meant by “with typically 120 -150 ewes in lactation contemporarily”?

Lines 149-156: I recommend providing additional methodological details to improve clarity and reproducibility. (i) Specifically, for soil sampling, please clarify how random sampling points were selected, indicate the sampling depth, and specify whether samples were collected from bulk soil or rhizospheric zones. (ii) Additionally, the rationale for uneven soil sample numbers across seasons should be explained. (iii) For both soil and fecal samples, please clarify whether biological or technical replicates were included. (iv) It would also strengthen the manuscript to briefly justify the choice of lactating ewes as the study population. (v) In line 155, “transfer” should be corrected to “transferred”.

Line 171: The Methods state that forward reads were truncated at 300 bp; since this corresponds to the full sequenced read length, it might be clearer to state that the forward reads were retained at full length, and truncation was applied only to reverse reads at 280 bp.

Line 181: Please provide the version of the vegan package as well as the versions of all other R packages used in the analyses.

Lines 183-185: The manuscript indicates that GRAZED and NGRAZED soils were compared using a paired t-test. However, the experimental design does not clearly describe a true pairing structure between samples. Since a paired t-test assumes a natural one-to-one correspondence between observations, clarification of the pairing rationale is needed. If samples were independent, an unpaired t-test may have been more appropriate.

Lines 188-191: The Kruskal–Wallis test was used to assess associations between categorical groups (ANIMAL, GRAZED, NGRAZED) and alpha diversity estimates among the four seasons. However, it is unclear whether season was incorporated as an independent factor in the analysis. I kindly suggest clarifying the statistical modeling approach to handle season.

Lines194-197: It is unclear whether the assumptions of PERMANOVA, particularly the homogeneity of group dispersions, were assessed. Differences in dispersion can bias significance. I suggest clarifying whether dispersion was checked.

Lines 198-203: The manuscript reports differential abundance analysis using DESeq2 with significance defined by both FDR ≤ 0.05 and fold-change thresholds (>1.5 or < -1.5), and additionally applies Bonferroni-adjusted p-value cutoffs that differ between soil (≤ 0.01) and ANIMAL samples (≤ 1×10⁻²⁰). The rationale for using both FDR and Bonferroni corrections, as well as the extremely stringent threshold for the ANIMAL group, is unclear. I suggest clarifying the justification for these choices and ensuring consistency in multiple-testing correction, so that the criteria for identifying significant taxa are transparent and biologically interpretable.

Line 208: In the Results section, the authors report summary statistics for soil physico-chemical parameters; however, the individual replicate values are not provided. Given the small sample size (n = 5 per area), access to raw data (e.g., in supplementary material) would improve transparency.

Table 1: Please clarify the meaning of the parenthetical values in the table and explain their absence for the clay, silt, and sand measurements.

Line 213: Please write out "Soil Organic Matter (SOM)" in full the first time it is mentioned, and subsequently use the abbreviation "SOM" if needed.

Line 219: Numerical data summarizing the distribution of ASVs by group at the phylum, family, and genus levels are not provided. Providing such counts or relative abundances in a supplementary table would greatly improve transparency and allow readers to evaluate the contribution of dominant and rare taxa to observed patterns. I recommend that the authors provide a detailed table or supplementary dataset summarizing ASV counts or relative abundances by group and season.

Line 272: The manuscript reports alpha diversity results using Shannon and Chao1 indices by some key values, but the individual numerical values for each sample are not provided. I recommend that the authors provide the full set of alpha diversity estimates per sample, along with the corresponding group and season information.

Table 2: The table presents PERMANOVA results in a raw-output style, which may be difficult for some readers to interpret. I suggest revising the table title to clearly indicate what is being tested and how. Additionally, consider adding brief footnotes explaining the columns (e.g., R², Residual), highlighting significant results, and formatting p-values and effect sizes for readability.

Lines 306-313: The results report differential abundance using only the most significant non-overlapping ASVs shown at the Family level. I recommend that the authors provide the full set of ASV-level results, including all significant taxa across seasons, and clarify the choice of thresholds.

Lines 365-367: In the Discussion, the authors state that similar physico-chemical properties between grazed and non-grazed soils support the reliability of subsequent microbial analyses, while this may be overstated, because this claim is true for bulk soil characteristics, the depth and sampling compartment (bulk vs. rhizosphere) were not specified. Given that sheep interact mainly with aboveground biomass and roots, soil microbial communities in the root zone may differ even when bulk properties are similar. I recommend clarifying the sampling depth and soil compartment, and discussing how plant-soil interactions and grazing could influence microbial composition, to contextualize links between sheep gut microbiota and soil communities more accurately.

Line 419: In the statement that “grazing appears to inhibit dominant groups such as Cyanobacteria” , the term “inhibit” is strong and involve a mechanistic investigation. The study reports relative abundances, and causation or direct inhibition cannot be inferred from observational data alone. I recommend rephrasing with more cautious descriptive terms, for example, indicating that grazing can negatively influence the abundance of … , so just a potential effect not a confirmed causation of a mechanistic phenomenon.

Lines 543-544: The statement that differential abundance of ASVs between grazed and ungrazed soils “supports the hypothesis of fecal microbiota being transferred to the soil” may overstate the findings. The data show associations between grazing and soil microbial composition, but direct evidence of microbial transfer from feces to soil was not measured as it requires tracking approach. I recommend rephrasing to more cautious statement, such as indicating that observed differences are consistent with a potential influence of fecal microbiota or that grazing may alter soil microbial communities, potentially including contributions from fecal input. please see: https://doi.org/10.1371/journal.pone.0316616

Line 560: The study’s limitations should be acknowledged, please state them in the conclusion. For example, repeated cross-sectional design instead of true longitudinal study, which limits the causality inference; only lactating ewes were sampled, and soil depth and compartment (bulk vs. rhizosphere) were not specified, which may affect links between gut and soil microbiota. The observational design precludes causal inferences between grazing, animal microbiota, and soil microbial changes. Additionally, differences in vegetation structure and seasonal variability may independently influence soil microbial communities.

Finally, I recommend that the authors make the raw sequencing data and ASV tables publicly available in a recognized repository to ensure transparency and reproducibility.

**********

6. PLOS authors have the option to publish the peer review history of their article (what does this mean?). If published, this will include your full peer review and any attached files.

If you choose “no”, your identity will remain anonymous but your review may still be made public.

**Do you want your identity to be public for this peer review?**  For information about this choice, including consent withdrawal, please see our Privacy Policy.

Reviewer #1: No

Reviewer #2: **Yes:** Houda Zouagui

**********

[NOTE: If reviewer comments were submitted as an attachment file, they will be attached to this email and accessible via the submission site. Please log into your account, locate the manuscript record, and check for the action link "View Attachments". If this link does not appear, there are no attachment files.]

To ensure your figures meet our technical requirements, please review our figure guidelines: https://journals.plos.org/plosone/s/figures

You may also use PLOS’s free figure tool, NAAS, to help you prepare publication quality figures: https://journals.plos.org/plosone/s/figures#loc-tools-for-figure-preparation.

NAAS will assess whether your figures meet our technical requirements by comparing each figure against our figure specifications.

# Author comment: Author response to Decision Letter 1

**DOI:** 10.1371/journal.pone.0352436.r002

8 Apr 2026

We would like to thank the Editor for considering our manuscript for publication and the Reviewers for their careful evaluation of our work, as well as for their constructive comments and suggestions. We have revised the manuscript accordingly and believe that these changes have improved its clarity and overall quality. Below, we provide a detailed, point-by-point response to each comment.

Reviewer #1: The manuscript is very interesting and well written, all the research points are well investigated and discussed. The topic is new and relevant and I only have some small format issues to present.

The main issue is the quality of the figures that seems low, can you please check? In the taxa abundance bar plots I suggest to keep in the legend only the taxa that are clearly visible in the figures to reduce the use of similar colors and I personally would avoid manteining taxa identified by a code (eg. Family XI in Fig.3 can be eliminated, also considering that is not discussed, as well as Clostridiales_vadinBB60_group can be called simply Clostridiales).

A: Thank you for your comments. Regarding the quality of the figures, we would like to clarify that all figures meet the journal’s requirement of at least 300 dpi resolution. Nevertheless, we carefully rechecked the files to ensure optimal quality in the submitted version.

Concerning the taxa abundance bar plots, we already applied a filtering criterion to improve readability: only phyla, families, and genera with relative abundance higher than 3% were included in the plots. We chose this threshold as a compromise between clarity and information content. Increasing this cutoff further would substantially reduce the amount of information conveyed by the figures.

Line 83 pg. 4: Please note that it should be bacterial and fungal taxa and not bacteria and fungi taxa.

A: Changed (L88).

Line 154 pg. 7: Please check the total number of soil samples.

A: Checked and confirmed.

Line 180 pg. 8: Please specify which version of Silva has been used.

A: SILVA version was added (L200).

Lines 233-235 pg. 11: Please rephrase for better clarity and fluency.

A: Rephrased (L274)

Lines 236-239: It seems to me that these changes are mainly in NGRAZED, correct? If so, please specify.

A: Authors checked and confirm the sentence.

Lines 247-249: Please rephrase for better clarity and fluency.

A: Rephrased (L289-290)

Line 263 pg. 12: Which is the difference between Ruminococcaceae_UCG.005 and Ruminococcaceae_UCG.010? I suggest to you to consider both taxa as Ruminococcaceae since there are not reported in the text information on their taxonomic difference.

A: Modified L307-306.

Lines 287-289 pg. 13: Can you please explain why PCA values are so small? It is because of the high variability in animal samples that is cited on lines 296-300?

A: Thank you for this comment. The relatively low percentage of variance explained by the first axes of the PCoA reflects the high compositional complexity and variability typical of microbiome datasets. In such datasets, community differences are often distributed across many dimensions rather than concentrated in the first few axes. The combined dataset including both sheep fecal samples and soil samples surely amplify the heterogeneous microbial communities. This increases overall beta-diversity and spreads the variance across multiple coordinates, resulting in lower percentages explained by the first axes. We have clarified this point in the revised manuscript (lines 351) to better explain that the low variance values are expected in highly diverse microbial datasets and do not affect the interpretation of the observed clustering patterns.

Reviewer #2: This manuscript investigates the relationship between sheep gut microbiota and soil microbial communities under grazing management across seasons, using fecal sampling from lactating ewes and soil sampling from grazed and ungrazed areas. The study applies 16S rRNA sequencing, diversity analyses, and differential abundance testing to explore how grazing influences microbial composition. The topic is timely and the dataset valuable; however, several aspects of the experimental design, statistical analysis, and interpretation of results warrant clarification. In the following comments, I provide specific suggestions on methodology, data presentation, and discussion points to improve transparency, rigor, and interpretability of the study.

Lines 53 – 56: I recommend explicitly referring to gut microbiota (or fecal microbiota, as appropriate) to improve conceptual clarity, be more specific, and avoid ambiguity.

A: Modified (L54)

Line 59: Please, consider rephrasing. I suggest “Collection of post mortem gut contents and mucosal scrapings, which precludes longitudinal studies (ref); endoscopic or biopsy-based sampling, which is technically challenging in large animals and often requires anesthesia or surgical intervention”

A: Modified (L61-64).

Line 66: As fecal sampling does not fully mirror the rumen microbial community and primarily reflects the distal gut environment, presenting it as a complete proxy for the entire gastrointestinal microbiota may be overstated. I recommend softening the statement to acknowledge this limitation. For example, fecal microbiota analyses can provide valuable insights into aspects of animal health.

A: Pointed out (L71).

Lines 89-90: The statement “extensive sheep grazing may represent a valuable approach that addresses all these concerns” may be somewhat overstated. While sheep grazing can contribute to sustainability, animal welfare, and mitigation of rural abandonment, it may be more accurate to frame these outcomes as potential benefits rather than definitive solutions. Given the scope of the present study, causal relationships between grazing and these broader socio-ecological outcomes cannot be established. I suggest moderating the language to reflect contribution or association rather than direct resolution.

A: We agree that the original wording could be interpreted as overstating the role of extensive sheep grazing. The sentence has been revised to moderate the claim and to emphasize its potential contribution rather than a definitive solution.

Line 91: The study design appears to be repeated cross-sectional rather than a true longitudinal setup, as different individuals were sampled across seasons rather than following the same animals longitudinally. I recommend stating this explicitly in the abstract, Methods section, Discussion, and Conclusion to avoid ambiguity. Additionally, the implications of this design should be acknowledged, as seasonal differences cannot be interpreted as within-individual temporal changes but rather as population-level trends. Clarifying this distinction would strengthen the methodological transparency of the study.

A: Modified in the whole manuscript and clarified in all sections.

Lines 110-111: I suggest reformulating “with a Ap-Bt-2Btk horizons succession” to “with an Ap-Bt-2Btk horizon sequence”.

A: Changed at L122.

Lines 112-113: Could the authors please clarify what is meant by “with typically 120 -150 ewes in lactation contemporarily”?

A: We have clarified this point in the revised manuscript (L124).

Lines 149-156: I recommend providing additional methodological details to improve clarity and reproducibility. (i) Specifically, for soil sampling, please clarify how random sampling points were selected, indicate the sampling depth, and specify whether samples were collected from bulk soil or rhizospheric zones. (ii) Additionally, the rationale for uneven soil sample numbers across seasons should be explained. (iii) For both soil and fecal samples, please clarify whether biological or technical replicates were included. (iv) It would also strengthen the manuscript to briefly justify the choice of lactating ewes as the study population. (v) In line 155, “transfer” should be corrected to “transferred”.

A: Authors answered below points by points and specified in the manuscript:

i) We have clarified that soil samples were collected from multiple locations distributed across the GRAZED and NGRAZED perimeters to capture spatial variability. Although a formal coordinate-based randomization scheme was not applied, sampling points were selected across the entire area while avoiding immediate proximity between collection points. Sampling depth was 10 cm from bulk soil. All these info were integrated in the manuscript.

(ii) The number of soil samples varied across seasons because the sampling effort was progressively expanded as additional funding became available during the study. A sentence was added at L169-170.

(iii) all samples represent biological replicates, with no technical replicates included.

(iv) they represent a metabolically homogeneous and management-relevant group within the flock.

(v) Corrected.

Line 171: The Methods state that forward reads were truncated at 300 bp; since this corresponds to the full sequenced read length, it might be clearer to state that the forward reads were retained at full length, and truncation was applied only to reverse reads at 280 bp.

A: We agree that the original wording could be unclear. The sentence has been revised to clarify that forward reads were retained at their full length, while truncation was applied only to reverse reads L191)

Line 181: Please provide the version of the vegan package as well as the versions of all other R packages used in the analyses.

A: All R packages versions have been added.

Lines 183-185: The manuscript indicates that GRAZED and NGRAZED soils were compared using a paired t-test. However, the experimental design does not clearly describe a true pairing structure between samples. Since a paired t-test assumes a natural one-to-one correspondence between observations, clarification of the pairing rationale is needed. If samples were independent, an unpaired t-test may have been more appropriate.

A: Authors thank the reviewer for this important comment and they have corrected this sentence in the revised manuscript by applying a two-tailed independent samples t-test (L204).

Lines 188-191: The Kruskal–Wallis test was used to assess associations between categorical groups (ANIMAL, GRAZED, NGRAZED) and alpha diversity estimates among the four seasons. However, it is unclear whether season was incorporated as an independent factor in the analysis. I kindly suggest clarifying the statistical modeling approach to handle season.

A: Authors agree that this was not clearly described in the original manuscript. The M&M section has now been revised to explicitly clarify that analyses were conducted independently for each season (L208-211).

Lines194-197: It is unclear whether the assumptions of PERMANOVA, particularly the homogeneity of group dispersions, were assessed. Differences in dispersion can bias significance. I suggest clarifying whether dispersion was checked.

A: We thank the reviewer for this important comment. We agree that assessing the homogeneity of multivariate dispersions is essential for a correct interpretation of PERMANOVA results.

We have now performed a PERMDISP analysis (using the betadisper function in the vegan R package) for each season. Results showed that dispersion was not significantly different among groups in Winter (p = 0.193) and Autumn (p = 0.459), whereas significant differences were detected in Spring (p = 0.008) and Summer (p = 0.001). The Materials and Methods and Results sections have been updated accordingly, as well as Discussion (e.g. L221, L575-579).

Lines 198-203: The manuscript reports differential abundance analysis using DESeq2 with significance defined by both FDR ≤ 0.05 and fold-change thresholds (>1.5 or < -1.5), and additionally applies Bonferroni-adjusted p-value cutoffs that differ between soil (≤ 0.01) and ANIMAL samples (≤ 1×10⁻²⁰). The rationale for using both FDR and Bonferroni corrections, as well as the extremely stringent threshold for the ANIMAL group, is unclear. I suggest clarifying the justification for these choices and ensuring consistency in multiple-testing correction, so that the criteria for identifying significant taxa are transparent and biologically interpretable.

A: Differential abundance was primarily assessed with false discovery rate (FDR) correction (Benjamini–Hochberg), and taxa were considered significant when FDR ≤ 0.05 together with a fold-change threshold (|log2FC| corresponding to >1.5 or < −1.5 to ensure biological relevance. The additional Bonferroni-adjusted p-value thresholds were applied only as a more conservative filter for reporting the most robust differences and for visualization purposes. Because the number of significant taxa detected in ANIMAL samples was substantially higher than in soil samples, a more stringent cutoff was applied to highlight the most strongly differentiated taxa and improve figure readability. To avoid confusion, we have now clarified the rationale for these thresholds in the Methods section (L225-232).

Line 208: In the Results section, the authors report summary statistics for soil physico-chemical parameters; however, the individual replicate values are not provided. Given the small sample size (n = 5 per area), access to raw data (e.g., in supplementary material) would improve transparency.

A: Data was included in Supplementary Table 1.

Table 1: Please clarify the meaning of the parenthetical values in the table and explain their absence for the clay, silt, and sand measurements.

Values in parentheses indicate standard deviations. Soil texture was determined on a pooled sample obtained by bulking all individual samples, and the analysis was performed in laboratory duplicate. Therefore, a single value without standard deviation is reported for texture. This information has now been added to the manuscript (L245).

Line 213: Please write out "Soil Organic Matter (SOM)" in full the first time it is mentioned, and subsequently use the abbreviation "SOM" if needed.

A: Changed.

Line 219: Numerical data summarizing the distribution of ASVs by group at the phylum, family, and genus levels are not provided. Providing such counts or relative abundances in a supplementary table would greatly improve transparency and allow readers to evaluate the contribution of dominant and rare taxa to observed patterns. I recommend that the authors provide a detailed table or supplementary dataset summarizing ASV counts or relative abundances by group and season.

A: We have provided a supplementary table containing, for each sample, the ASV-level relative abundances together with the corresponding alpha diversity indices (observed richness S.obs and Shannon index shannon), as well as sample metadata including group (GRAZED, NGRAZED, ANIMAL) and season (AUT, WIN, SPR, SUM). This table allows readers to examine the full set of alpha diversity estimates and evaluate the contribution of each ASV to community composition across groups and seasons.

Line 272: The manuscript reports alpha diversity results using Shannon and Chao1 indices by some key values, but the individual numerical values for each sample are not provided. I recommend that the authors provide the full set of alpha diversity estimates per sample, along with the corresponding group and season information.

A: We have provided a supplementary table containing all the requested information.

Table 2: The table presents PERMANOVA results in a raw-output style, which may be difficult for some readers to interpret. I suggest revising the table title to clearly indicate what is being tested and how. Additionally, consider adding brief footnotes explaining the columns (e.g., R², Residual), highlighting significant results, and formatting p-values and effect sizes for readability.

A: Modifications have been done to better interpret results.

Lines 306-313: The results report differential abundance using only the most significant non-overlapping ASVs shown at the Family level. I recommend that the authors provide the full set of ASV-level results, including all significant taxa across seasons, and clarify the choice of thresholds.

A: Details on thresholds have been added, as requested above. S4 Table reports now the list of significant taxa, as the reviewer suggests.

Lines 365-367: In the Discussion, the authors state

Attachment Submitted filename: Response_to_reviewers.docx

# Peer review: Decision Letter 1

**DOI:** 10.1371/journal.pone.0352436.r003

**Authors:** Raffaella Balestrini, Raffaella Balestrini

17 May 2026

<div>PONE-D-25-59668R1

Seasonal dynamics in sheep fecal microbiome and soil bacterial communities under grazing management

PLOS One

Dear Dr. Fabbri,

Thank you for submitting your manuscript to PLOS ONE. After careful consideration, we feel that it has merit but does not fully meet PLOS ONE’s publication criteria as it currently stands. Therefore, we invite you to submit a revised version of the manuscript that addresses the points raised during the review process.

Please submit your revised manuscript by Jul 01 2026 11:59PM. If you will need more time than this to complete your revisions, please reply to this message or contact the journal office at plosone@plos.org. When you're ready to submit your revision, log on to https://www.editorialmanager.com/pone/ and select the 'Submissions Needing Revision' folder to locate your manuscript file.

Please include the following items when submitting your revised manuscript:

- A letter that responds to each point raised by the academic editor and reviewer(s). You should upload this letter as a separate file labeled 'Response to Reviewers'.
- A marked-up copy of your manuscript that highlights changes made to the original version. You should upload this as a separate file labeled 'Revised Manuscript with Track Changes'.
- An unmarked version of your revised paper without tracked changes. You should upload this as a separate file labeled 'Manuscript'.

If you would like to make changes to your financial disclosure, please include your updated statement in your cover letter. Guidelines for resubmitting your figure files are available below the reviewer comments at the end of this letter.

If applicable, we recommend that you deposit your laboratory protocols in protocols.io to enhance the reproducibility of your results. Protocols.io assigns your protocol its own identifier (DOI) so that it can be cited independently in the future. For instructions see: https://journals.plos.org/plosone/s/submission-guidelines#loc-laboratory-protocols. Additionally, PLOS ONE offers an option for publishing peer-reviewed Lab Protocol articles, which describe protocols hosted on protocols.io. Read more information on sharing protocols at https://plos.org/protocols?utm_medium=editorial-email&utm_source=authorletters&utm_campaign=protocols.

As the corresponding author, your ORCID iD is verified in the submission system and will appear in the published article. PLOS supports the use of ORCID, and we encourage all coauthors to register for an ORCID iD and use it as well. Please encourage your coauthors to verify their ORCID iD within the submission system before final acceptance, as unverified ORCID iDs will not appear in the published article. *Only*  the individual author can complete the verification step; PLOS staff *cannot*  verify ORCID iDs on behalf of authors.

We look forward to receiving your revised manuscript.

Kind regards,

Raffaella Balestrini

Academic Editor

PLOS One

Journal Requirements:

If the reviewer comments include a recommendation to cite specific previously published works, please review and evaluate these publications to determine whether they are relevant and should be cited. There is no requirement to cite these works unless the editor has indicated otherwise.

Please review your reference list to ensure that it is complete and correct. If you have cited papers that have been retracted, please include the rationale for doing so in the manuscript text, or remove these references and replace them with relevant current references. Any changes to the reference list should be mentioned in the rebuttal letter that accompanies your revised manuscript. If you need to cite a retracted article, indicate the article’s retracted status in the References list and also include a citation and full reference for the retraction notice.

[Note: HTML markup is below. Please do not edit.]

Reviewers' comments:

Reviewer's Responses to Questions

**Comments to the Author**

1. If the authors have adequately addressed your comments raised in a previous round of review and you feel that this manuscript is now acceptable for publication, you may indicate that here to bypass the “Comments to the Author” section, enter your conflict of interest statement in the “Confidential to Editor” section, and submit your "Accept" recommendation.

Reviewer #2: (No Response)

**********

2. Is the manuscript technically sound, and do the data support the conclusions?

The manuscript must describe a technically sound piece of scientific research with data that supports the conclusions. Experiments must have been conducted rigorously, with appropriate controls, replication, and sample sizes. The conclusions must be drawn appropriately based on the data presented.

Reviewer #2: Yes

**********

3. Has the statistical analysis been performed appropriately and rigorously?

Reviewer #2: Yes

**********

4. Have the authors made all data underlying the findings in their manuscript fully available?

The PLOS Data policy requires authors to make all data underlying the findings described in their manuscript fully available without restriction, with rare exception (please refer to the Data Availability Statement in the manuscript PDF file). The data should be provided as part of the manuscript or its supporting information, or deposited to a public repository. For example, in addition to summary statistics, the data points behind means, medians and variance measures should be available. If there are restrictions on publicly sharing data—e.g. participant privacy or use of data from a third party—those must be specified.

Reviewer #2: Yes

**********

5. Is the manuscript presented in an intelligible fashion and written in standard English?

PLOS ONE does not copyedit accepted manuscripts, so the language in submitted articles must be clear, correct, and unambiguous. Any typographical or grammatical errors should be corrected at revision, so please note any specific errors here.

Reviewer #2: Yes

**********

6. Review Comments to the Author

Please use the space provided to explain your answers to the questions above. You may also include additional comments for the author, including concerns about dual publication, research ethics, or publication ethics. (Please upload your review as an attachment if it exceeds 20,000 characters)

Reviewer #2: I would like to thank the authors for carefully and thoroughly addressing all the comments, and I congratulate them on the high quality of their revision and the great effort. I have only a few minor remarks that the authors should address.

Line 59: Please replace the term “longitudinal studies” with “over‑time studies” to maintian consistency in terminology and methodology. Please also review the corresponding citation if appropriate.

Please explicitly refer to the study design as “repeated cross‑sectional” rather than merely “cross‑sectional” wherever it appears in the manuscript, as the “repeated cross‑sectional” designation clearly reflects an additional parameter (seasons, in this case).

Line 113: I suggested reformulating “with a Ap-Bt-2Btk horizons succession” to “with an Ap–Bt–2Btk horizon sequence” because the latter phrasing is more consistent with standard soil-profile terminology, where “horizon sequence” is conventionally used to describe the vertical arrangement of diagnostic horizons. Please update the expression to improve clarity and terminological accuracy.

In my previous comment, I requested additional methodological clarifications to improve transparency and reproducibility, including clarification of whether biological or technical replicates were included for soil and fecal samples, and a brief justification for selecting lactating ewes as the study population. The authors have addressed these points in their response to reviewers, but the corresponding explanations have not yet been incorporated into the manuscript text. Please add clear, concise statements in the Methods (or relevant section) explicitly stating the use of biological replicates for soil and fecal samples, and the rationale for choosing lactating ewes as the study population.

**********

7. PLOS authors have the option to publish the peer review history of their article (what does this mean?). If published, this will include your full peer review and any attached files.

If you choose “no”, your identity will remain anonymous but your review may still be made public.

**Do you want your identity to be public for this peer review?**  For information about this choice, including consent withdrawal, please see our Privacy Policy.

Reviewer #2: **Yes:** Houda Zouagui

**********

[NOTE: If reviewer comments were submitted as an attachment file, they will be attached to this email and accessible via the submission site. Please log into your account, locate the manuscript record, and check for the action link "View Attachments". If this link does not appear, there are no attachment files.]

To ensure your figures meet our technical requirements, please review our figure guidelines: https://journals.plos.org/plosone/s/figures

You may also use PLOS’s free figure tool, NAAS, to help you prepare publication quality figures: https://journals.plos.org/plosone/s/figures#loc-tools-for-figure-preparation.

NAAS will assess whether your figures meet our technical requirements by comparing each figure against our figure specifications.

# Author comment: Author response to Decision Letter 2

**DOI:** 10.1371/journal.pone.0352436.r004

21 May 2026

We would like to thank the Editor for considering our manuscript for publication and the Reviewer for their careful evaluation of our work, as well as for the constructive comments and suggestions. We have revised the manuscript accordingly and believe that these changes have improved its clarity and overall quality. Below, we provide a detailed, point-by-point response to each comment.

Reviewer #2: I would like to thank the authors for carefully and thoroughly addressing all the comments, and I congratulate them on the high quality of their revision and the great effort. I have only a few minor remarks that the authors should address.

Line 59: Please replace the term “longitudinal studies” with “over‑time studies” to maintian consistency in terminology and methodology. Please also review the corresponding citation if appropriate.

A: Changed (L59)

Please explicitly refer to the study design as “repeated cross‑sectional” rather than merely “cross‑sectional” wherever it appears in the manuscript, as the “repeated cross‑sectional” designation clearly reflects an additional parameter (seasons, in this case).

A: The term has been changed in the whole manuscript: L40, L385

Line 113: I suggested reformulating “with a Ap-Bt-2Btk horizons succession” to “with an Ap–Bt–2Btk horizon sequence” because the latter phrasing is more consistent with standard soil-profile terminology, where “horizon sequence” is conventionally used to describe the vertical arrangement of diagnostic horizons. Please update the expression to improve clarity and terminological accuracy.

A: The term “Ap-Bt-2Btk horizons succession“ has been updated (L114).

In my previous comment, I requested additional methodological clarifications to improve transparency and reproducibility, including clarification of whether biological or technical replicates were included for soil and fecal samples, and a brief justification for selecting lactating ewes as the study population. The authors have addressed these points in their response to reviewers, but the corresponding explanations have not yet been incorporated into the manuscript text. Please add clear, concise statements in the Methods (or relevant section) explicitly stating the use of biological replicates for soil and fecal samples, and the rationale for choosing lactating ewes as the study population.

A: Clarifications regarding the use of biological replicates and the rationale for selecting lactating ewes have been added to the Methods section (L155–158).

Attachment Submitted filename: Response_to_reviewers_auresp_2.docx

# Editor report: Decision Letter 2

**DOI:** 10.1371/journal.pone.0352436.r005

**Authors:** Raffaella Balestrini, Raffaella Balestrini, Raffaella Balestrini

11 Jun 2026

Seasonal dynamics in sheep fecal microbiome and soil bacterial communities under grazing management

PONE-D-25-59668R2

Dear Dr. Fabbri,

We’re pleased to inform you that your manuscript has been judged scientifically suitable for publication and will be formally accepted for publication once it meets all outstanding technical requirements.

Within one week, you’ll receive an e-mail detailing the required amendments. When these have been addressed, you’ll receive a formal acceptance letter and your manuscript will be scheduled for publication.

An invoice will be generated when your article is formally accepted. Please note, if your institution has a publishing partnership with PLOS and your article meets the relevant criteria, all or part of your publication costs will be covered. Please make sure your user information is up-to-date by logging into Editorial Manager at Editorial Manager® and clicking the ‘Update My Information' link at the top of the page. For questions related to billing, please contact billing support.

If your institution or institutions have a press office, please notify them about your upcoming paper to help maximize its impact. If they’ll be preparing press materials, please inform our press team as soon as possible -- no later than 48 hours after receiving the formal acceptance. Your manuscript will remain under strict press embargo until 2 pm Eastern Time on the date of publication. For more information, please contact onepress@plos.org.

Kind regards,

Raffaella Balestrini

Academic Editor

PLOS One

Additional Editor Comments (optional):

Reviewers' comments:

# Editor report: Acceptance letter

**DOI:** 10.1371/journal.pone.0352436.r006

**Authors:** Raffaella Balestrini, Raffaella Balestrini, Raffaella Balestrini

PONE-D-25-59668R2

PLOS One

Dear Dr. Fabbri,

I'm pleased to inform you that your manuscript has been deemed suitable for publication in PLOS One. Congratulations! Your manuscript is now being handed over to our production team.

At this stage, our production department will prepare your paper for publication. This includes ensuring the following:

* All references, tables, and figures are properly cited

* All relevant supporting information is included in the manuscript submission,

* There are no issues that prevent the paper from being properly typeset

You will receive further instructions from the production team, including instructions on how to review your proof when it is ready. Please keep in mind that we are working through a large volume of accepted articles, so please give us a few days to review your paper and let you know the next and final steps.

Lastly, if your institution or institutions have a press office, please let them know about your upcoming paper now to help maximize its impact. If they'll be preparing press materials, please inform our press team within the next 48 hours. Your manuscript will remain under strict press embargo until 2 pm Eastern Time on the date of publication. For more information, please contact onepress@plos.org.

You will receive an invoice from PLOS for your publication fee after your manuscript has reached the completed accept phase. If you receive an email requesting payment before acceptance or for any other service, this may be a phishing scheme. Learn how to identify phishing emails and protect your accounts at https://explore.plos.org/phishing.

If we can help with anything else, please email us at customercare@plos.org.

Thank you for submitting your work to PLOS ONE and supporting open access.

Kind regards,

PLOS ONE Editorial Office Staff

on behalf of

Dr Raffaella Balestrini

Academic Editor

PLOS One
